# Lesion location changes the association between brain excitability and the performance of a short-term visuomotor adaptation task post-stroke

**DOI:** 10.1093/braincomms/fcaf430

**Published:** 2025-10-31

**Authors:** Bernat De Las Heras, Lynden Rodrigues, Jacopo Cristini, Kevin Moncion, Numa Dancause, Alexander Thiel, Jodi D Edwards, Janice J Eng, Ada Tang, Marc Roig

**Affiliations:** Memory and Motor Rehabilitation Laboratory (MEMORY-LAB), Feil and Oberfeld Research Centre, Jewish Rehabilitation Hospital, Montreal Center for Interdisciplinary Research in Rehabilitation (CRIR), Laval, QC H7V 1R2, Canada; School of Physical and Occupational Therapy, Faculty of Medicine, McGill University, Montréal, QC, Canada; Memory and Motor Rehabilitation Laboratory (MEMORY-LAB), Feil and Oberfeld Research Centre, Jewish Rehabilitation Hospital, Montreal Center for Interdisciplinary Research in Rehabilitation (CRIR), Laval, QC H7V 1R2, Canada; School of Physical and Occupational Therapy, Faculty of Medicine, McGill University, Montréal, QC, Canada; Memory and Motor Rehabilitation Laboratory (MEMORY-LAB), Feil and Oberfeld Research Centre, Jewish Rehabilitation Hospital, Montreal Center for Interdisciplinary Research in Rehabilitation (CRIR), Laval, QC H7V 1R2, Canada; School of Physical and Occupational Therapy, Faculty of Medicine, McGill University, Montréal, QC, Canada; Memory and Motor Rehabilitation Laboratory (MEMORY-LAB), Feil and Oberfeld Research Centre, Jewish Rehabilitation Hospital, Montreal Center for Interdisciplinary Research in Rehabilitation (CRIR), Laval, QC H7V 1R2, Canada; School of Physical and Occupational Therapy, Faculty of Medicine, McGill University, Montréal, QC, Canada; Faculté de Médecine, Département de Neurosciences, Université de Montréal, Montréal, QC, Canada; Department of Neurology and Neurosurgery, Faculty of Medicine, McGill University, Montréal, QC, Canada; School of Epidemiology and Public Health, University of Ottawa Heart Institute, Ottawa, ON, Canada; Centre for Aging SMART at Vancouver Coastal Health and Department of Physical Therapy, University of British Columbia, Vancouver, BC, Canada; School of Rehabilitation Science, Faculty of Health Sciences, McMaster University, Hamilton, ON, Canada; Memory and Motor Rehabilitation Laboratory (MEMORY-LAB), Feil and Oberfeld Research Centre, Jewish Rehabilitation Hospital, Montreal Center for Interdisciplinary Research in Rehabilitation (CRIR), Laval, QC H7V 1R2, Canada; School of Physical and Occupational Therapy, Faculty of Medicine, McGill University, Montréal, QC, Canada

**Keywords:** transcranial magnetic stimulation, motor skill learning, GABA, glutamate, movement evoked potential

## Abstract

The capacity to reacquire motor skills lost after a stroke is crucial to promote upper-limb motor recovery, but the impact of lesion location on motor skill acquisition and the underlying neurophysiological mechanisms remain uncertain. Cortico-spinal excitability, measured with transcranial magnetic stimulation, provides information about the functional integrity of the cortico-spinal tract, a structure essential for upper-limb motor function and recovery post-stroke. Cortico-spinal excitability is a biomarker that has been used to predict upper-limb motor recovery and guide interventions post-stroke. We investigated associations between different cortico-spinal excitability measures of facilitation and inhibition and the capacity to improve the performance of a short-term visuomotor adaptation task that required accurate force modulation with the most affected hand in 103 individuals with either cortical (*n* = 34) or subcortical (*n* = 69) lesions. Cortico-spinal excitability measures included resting motor threshold, resting and active excitability derived from the amplitude of motor evoked potentials, cortical silent period, as well as intracortical facilitation and short intracortical inhibition. Both lesion groups showed similar rates of motor skill performance but individuals with subcortical lesions exhibited more impairment in the most affected hand and lower excitability in the ipsilesional hemisphere inferred from a reduced amplitude of motor evoked potentials elicited during an active muscle contraction. Exploratory analyses revealed that upper-limb impairments and reductions in active and resting excitability in the ipsilesional hemisphere were exacerbated in individuals with subcortical lesions affecting the cortico-spinal tract. In individuals with cortical lesions, better motor skill performance was associated with lower motor thresholds (β = −0.25, 95% CI [−0.47, −0.03]; *P* = 0.024) and higher intracortical inhibition (β = −3.93, 95% CI [−6.89, −0.98]; *P* = 0.011) in the ipsilesional hemisphere. In contrast, in individuals with subcortical lesions motor skill performance was associated with smaller motor evoked potentials (β = −4.46, 95% CI [−8.54, −0.38]; *P* = 0.033), less intracortical inhibition (β = 3.45, 95% CI [0.34,6.56]; *P* = 0.030) and higher facilitation (β = 1.34, 95% CI [0.15,2.54]; *P* = 0.028) in the ipsilesional hemisphere. Associations with intracortical inhibition and facilitation in the subcortical group were driven primarily by observations from individuals with lesions affecting the cortico-spinal tract. Importantly, no associations were found in the contralesional hemisphere. Reinforcing the existence of lesion-specific neurophysiological reorganization patterns, individuals with cortical and subcortical lesions show divergent associations between cortico-spinal excitability and the capacity to improve the performance of a short-term visuomotor adaptation task. The use of cortico-spinal excitability as a biomarker to guide motor recovery interventions such as non-invasive brain stimulation or to predict upper-limb recovery post-stroke should consider lesion location.

## Teaser

De Las Heras et al. report that associations between different measures of cortico-spinal excitability and the performance of an upper-limb visuomotor task that requires accurate force modulation with the most affected hand are diametrically opposed in patients with cortical and subcortical brain lesions.

## Graphical Abstract

**Graphical Abstract:**
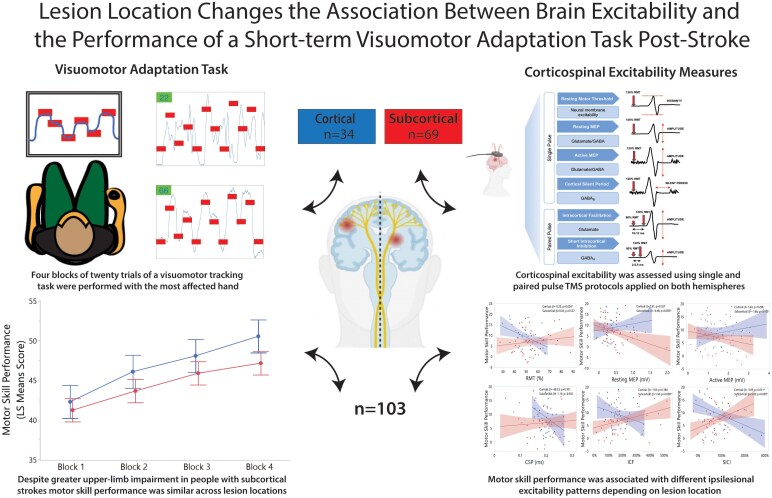

## Introduction

Stroke is a leading cause of disability worldwide, with up to 80% of patients experiencing upper-limb motor deficits in acute stages of recovery, and only around half achieving full upper-limb recovery six months after the cerebrovascular accident has occurred.^1^ Upper-limb motor impairments, which can lead to difficulties in the execution of motor skills that are essential for activities of daily living such as reaching or holding objects, can reduce functional independence and health-related quality of life.^2^

Although motor skill learning, which is the ability to acquire and retain motor skills,^3^ is distinct from motor recovery, a term that defines the restoration of pre-lesion motor performance and function,^4^ the two processes share neurophysiological mechanisms. Indeed, several studies have demonstrated that comparable changes in neuronal excitability can occur in response to motor skill learning and during motor recovery post-stroke.^5^ These studies support the view that the mechanisms underlying motor skill learning provide a substrate for motor recovery and thus could guide motor rehabilitation in these patients.^3^

Shared mechanisms supporting motor skill learning and stroke recovery have been well characterized in animal studies but are less understood in humans.^6^ This is in part because, while stroke lesions induced on animals can be rigorously localized and controlled, stroke lesions in humans have greater neuroanatomical variability. In addition, aspects such as age, previous neurological injury, comorbidities, and medication use, also contribute to increase the heterogeneity of clinical outcomes post-stroke in humans. Reducing this heterogeneity by controlling lesion-related characteristics could improve our understanding of the mechanisms underlying upper-limb recovery after stroke.^7^

Lesion location plays a fundamental role in determining both initial impairment and motor recovery after stroke.^8^ The ability to regain upper-limb motor function is strongly dependent on the functional integrity of the cortico-spinal tract (CST).^9^ Compared with lesions affecting cortical areas, subcortical lesions affecting the CST tend to result in larger impairments and poorer motor recovery.^8^ Individuals with subcortical lesions also tend to show a more widespread brain activation during upper-limb paretic movements,^10^ with more damage in the CST leading to increased recruitment of secondary motor networks.^11^

**Figure 1:**
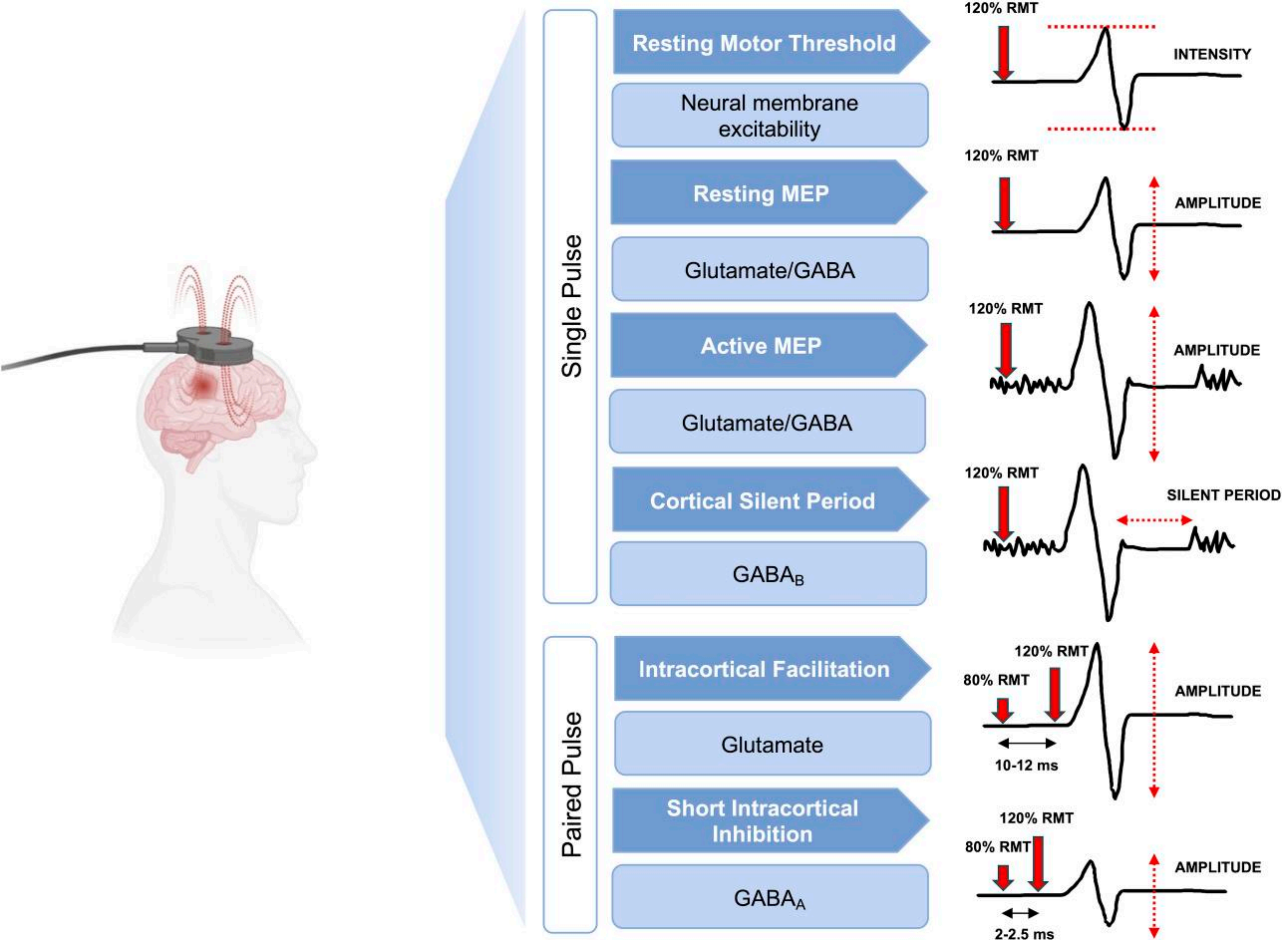
**Single and paired pulse transcranial magnetic stimulation protocols used to assess different cortico-spinal excitability (CSE) measures and their putative underlying mechanisms.** Resting Motor Threshold (RMT) is shown as a percentage of the stimulator output intensity needed to reach a consistent motor evoked potential (MEP) amplitude of 0.05 mV. It reflects neural membrane excitability, with lower RMT indicating higher CSE. Resting and active MEP amplitude measure the excitability of cortical and spinal projections influenced by excitatory (glutamate) and inhibitory (GABA) circuits, with larger MEP amplitudes indicating higher CSE. Cortical Silent Period (CSP) reflects GABA_B_ receptor-mediated inhibition, with longer CSP indicating greater inhibition. Intracortical Facilitation (ICF) measures facilitation mediated by NMDA receptors and glutamatergic neural activity, and SICI (Short Intracortical Inhibition) assesses inhibition mediated by GABA_A_ receptors. Larger ICF values indicate greater facilitation, while larger SICI values indicate reduced inhibition. GABA, gamma-aminobutyric acid; ms, milliseconds; NMDA, *N*-methyl-D-aspartate.

Transcranial magnetic stimulation (TMS) has been widely used to assess the functional integrity of the CST in stroke studies.^12^ While variability is substantial,^13^ cortico-spinal excitability (CSE) measures obtained with single-pulse TMS protocols such as reduced motor evoked potential (MEP) response, have shown certain capacity to predict upper-limb motor recovery post-stroke.^9^ Similarly, paired-pulse TMS protocols can estimate intracortical facilitation and inhibition mechanisms regulated by excitatory (glutamate) and inhibitory (γ-aminobutyric acid -GABA-) neurotransmitters^14^ involved in stroke recovery^5^ (Fig. 1). However, since CSE can change dynamically throughout stroke recovery,^15^ and the specific characteristics of the lesion increase inter-individual variability,^16^ considering these two factors is important.

Lesions involving cortical and subcortical brain structures lead to different CSE patterns^16^ and lesion location can affect the relationship between CSE and upper-limb motor impairment.^17^ Despite the importance of motor skill learning for upper-limb recovery,^18^ there is little information about the relationship between lesion location and the ability to perform motor skills after stroke.^19,20^ Even less is known about how lesion location influences the association between motor skill performance and different aspects of CSE. We investigated the influence of lesion location on the association between CSE and the capacity to improve the performance of an upper-limb motor skill across the stroke recovery continuum using a short-term visuomotor adaptation task. We hypothesized that, regardless of stage recovery, the associations between CSE and motor performance would differ between cortical and subcortical lesions.

## Materials and methods

### Experimental design

This observational study, which used baseline TMS data of participants enrolled in two registered randomized controlled trials (NCT03614585, NCT05076747), adheres to STROBE guidelines.^21^ In the first experimental session, in addition to demographic measures, stroke severity, cognitive function, upper-limb impairment and function were assessed (see clinical outcomes section for details). Motor skill performance was assessed with a short-term visuomotor adaptation task using the hand contralateral to the lesioned hemisphere.^22^ TMS was applied over the primary motor cortex (M1) of the ipsilesional and contralesional hemispheres to obtain different CSE measures (Fig. 1). The ethics board of the site (Centre de Recherche de Readaptation du Montréal, CRIR-1265-0817, CRIR-1310-0218) approved the study. Participants provided written consent prior to participation according to the Declaration of Helsinki.

### Participants

We included individuals with first-ever ischaemic or haemorrhagic stroke, from early subacute to chronic stages (7 days to 5 years post-stroke) of recovery, with no upper-limb musculoskeletal or neurological conditions other than stroke-related motor deficits, and no TMS contraindications.^23^ Lesion location was determined from the clinical reports of radiologists who analysed the CT/MRI scans obtained at admission in different hospitals (≤2 days after the stroke event) to identify the brain structures affected by the stroke. Radiological reports underwent thorough analysis by our team, which includes neurologists and neuroimaging experts with extensive experience in both human and non-human primate brain anatomy. For the primary analysis (see statistical analysis section), participants were classified as either subcortical or cortical. The subcortical group included individuals with lesions involving only subcortical regions and the cortical group individuals with lesions involving cortical regions (e.g. frontal, parietal, occipital) as well as those with combined cortical-subcortical lesions. This categorization has been used previously in both TMS^16,17,24^ and neuroimaging studies.^10,25^ To investigate the influence of CST integrity and, importantly, to confirm that our lesion categorization was accurate, we conducted an exploratory analysis dividing the subcortical group between participants with lesions affecting the structures of the CST (e.g. internal capsule, corona radiata) and participants with subcortical lesions outside the CST (e.g. basal ganglia, thalamus). Individuals with lesions located in the cerebellum or brainstem were excluded.

### Clinical outcomes

Stroke severity was assessed with the National Institutes of Health Stroke Scale (NIHSS),^26^ and cognitive status with the Montreal Cognitive Assessment (MoCA).^27^ The Chedoke-McMaster Stroke Assessment (CMSA),^28^ and the Box and Block Test (BBT)^29^ were used to assess upper-limb motor impairment and function, respectively. The CMSA is a screening and assessment tool utilized to measure physical impairment and activity of an individual following a stroke.^28^ The BBT is a test of unilateral gross manual dexterity, in which participants are instructed to move as many small wooden blocks as possible from one side of a partitioned box to the other within one minute using their affected and unaffected side.^29^

### Maximal voluntary contraction

Handgrip maximal voluntary contraction (MVC) force was assessed for both hands separately using a custom LabView (National Instruments, TX, USA) script. Holding a handgrip force response pad (TSD-121, Biopac, CA, USA) connected to a force transducer that converted grip force into a digital signal, participants comfortably seated viewed a slider on a 27-inch screen providing visual feedback on the force produced. They performed three 3-second MVCs with 30-second pauses and the highest MVC was recorded.^22^

### Short-term visuomotor adaptation task

**Figure 2:**
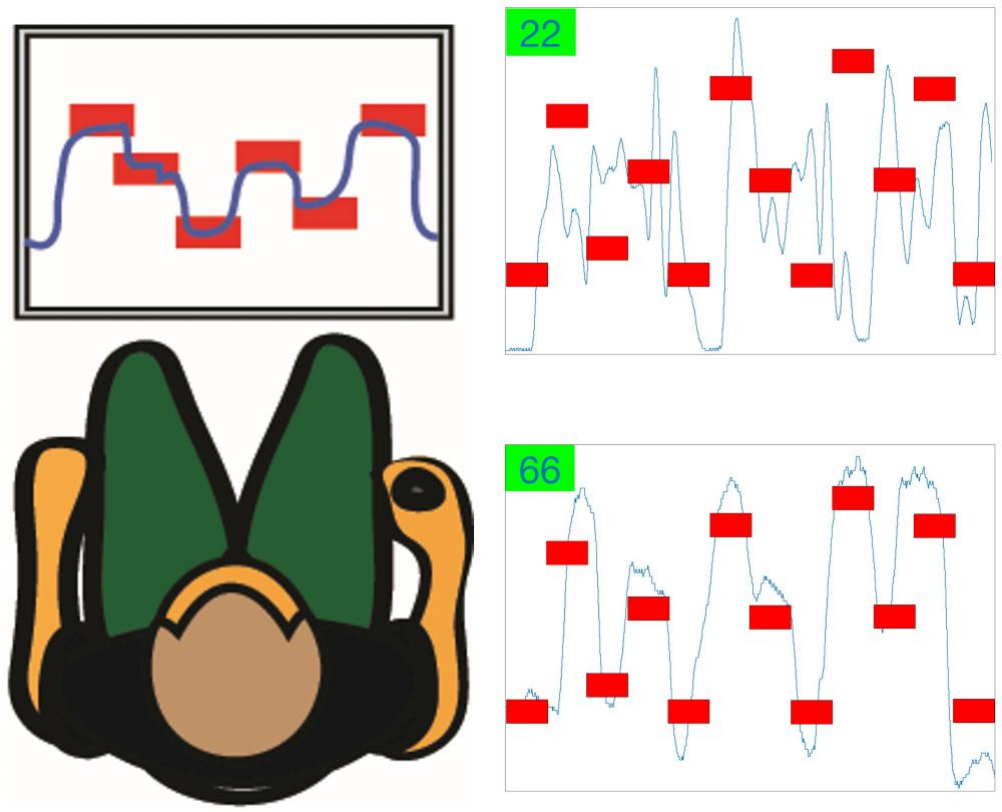
**Short-term visuomotor adaptation task used to assess motor skill performance**. The blue cursor crossed a 27-inch computer screen from left to right at a constant speed of 8 s/screen. Participants needed to apply or release grip force to adjust the blue cursor up or down in order reach the 12 red targets displayed horizontally at different heights. The force required to reach the highest target was ∼20% of MVC. The goal was to keep the cursor within the targets as much time as possible. Examples of two force traces demonstrating improvement from baseline (score = 22) to the best block of practice (score = 66) are presented.

To assess motor skill performance, participants completed a short-term visuomotor adaptation task that requires fine hand-grasping force modulation, using the same handgrip force response pad of the MVC assessment with the hand contralateral to the lesioned hemisphere.^22^ In short, participants were instructed to increase or release grip force to maintain the cursor within predefined target zones for as long as possible, while keeping a steady grip position (Fig. 2). The task, implemented via a custom MATLAB (Mathworks, MA, USA) script, recorded the time the cursor remained within the target zone for each trial and has been previously used in previous stroke studies.^22^

To minimize baseline differences, participants completed familiarization trials until they obtained a score >30 at least three times before practice. Practice consisted of 4 blocks of 20 trials each with 1–2 min of rest between blocks.^22^ Excluding familiarization, total practice time was 21.6 min. The score (0–100), which was visually presented at the end of each trial, was calculated as time on target divided by total time of each trial multiplied by 100. The more time the cursor was on target the higher the score (Fig. 2). The difference in mean scores from the first block to the best block of practice was used as a measure of motor skill performance.^22^

### Transcranial magnetic stimulation

Using neuronavigation (Brainsight, Rogue Research Inc, QC, Canada), we first co-registered the patients’ heads to a standard MRI template to guide TMS targeting. We used a mapping procedure that systematically elicited MEPs from M1 using stereotaxic navigation guided by a stimulation grid. The ‘hot spot’ was defined as the coil position that produced the largest and most consistent MEPs.^30^ TMS was applied using a 70-mm coil connected to two Magstim 200^2^ magnetic stimulators via a Bistim^2^ unit (Magstim, Whitland, Wales, UK). The coil handle pointed posterior-laterally at a 45° angle relative to the midsagittal plane, delivering posterior-anterior currents targeting the M1 representational area of the first dorsal interosseous muscle in both the ipsilesional and contralesional hemispheres.^31^

Electromyographic activity was recorded via two surface electrodes placed over the first dorsal interosseous ∼1 cm apart. Data was acquired at 2000 Hz through a CED Micro1401-4 unit, controlled by Signal software (CED, Cambridge, UK), with a gain of 300 and filtered using 10 Hz high-pass and 500 Hz low-pass filters. The level of background muscle activity was monitored continuously and trials with any excessive electromyographic (EMG) activity (>0.05 mV amplitude) 300 ms before stimulation were removed from the analysis. Different CSE measures were obtained using single- and paired-pulse TMS protocols, with pulses (inter-trial interval) delivered every 5 s to minimize potential repetitive TMS effects on MEP amplitude (Fig. 1).

### Cortico-spinal excitability

#### Single-pulse protocols

*Resting motor threshold (RMT):* defined as the minimum stimulation intensity required to elicit MEPs of >0.05 mV in at least 10 of 20 trials.^31^  *MEP amplitude:* assessed separately at rest and during an active muscle contraction sustained at 10% of the MVC. To assess active excitability, the LabView script used to measure MVC provided visual feedback while participants were asked to maintain the muscle contraction at the 10% MVC level. MEP amplitude was quantified by averaging the peak-to-peak amplitude from 60 stimulations (30 resting and 30 active) delivered 5 s apart at 120% RMT.^32^  *Cortical silent period (CSP)*: the CSP is a period of electrical silence in the EMG activity following an MEP during isotonic muscle contraction.^31^ CSP was extracted from 30 stimulations at 120% RMT during an active contraction. The EMG baseline signal amplitude 200 ms before stimulation was measured. The end of the MEP and the recovery of the voluntary EMG activity (i.e. two standard deviations above the mean baseline signal amplitude) marked the beginning and the end of the CSP, respectively.^22^ This approach to measure (absolute) CSP is preferred because excluding the MEP minimizes the influence of MEP excitatory mechanisms that differ from the inhibitory effects underlying CSP.^33^

#### Paired-pulse protocols

*Intracortical facilitation (ICF) and short intracortical inhibition (SICI):* measured using a conditioning pulse of 80% RMT followed by a suprathreshold pulse (120% RMT) delivered at rest after 10–12 (ICF) and 2–2.5 ms (SICI), respectively.^31^ To estimate facilitation and inhibition, the amplitude of the MEP elicited by the second conditioned pulse was normalized to the unconditioned test stimuli used to assess resting MEP amplitudes at 120% RMT.^22^ Normalized values are represented as percentages, with values >100% indicating ICF and values <100% indicating SICI. A total of 60 paired pulses (30 for ICF and 30 for SICI) were delivered with an interstimulus interval of 5 s.^31^

### Statistical analysis

Data were visually inspected with histograms and normal quantile plots, and the Shapiro-Wilk test was used to confirm normality of distribution for each variable. For the primary analysis, differences between cortical and subcortical groups in demographic and clinical variables were investigated using t-tests and Wilcoxon tests for continuous variables. Chi-square tests (X^2^) were used to compare these two groups in categorical variables.

Repeated linear mixed models (LMM) were used to assess differences between cortical and subcortical groups in motor skill performance. Scores of practice blocks (Block 1 to 4) were the dependent variable, and block, group, and their interaction, were treated as fixed effects, and participants as a random effect. Stroke severity (NIHSS), age, time since stroke (days), and handgrip MVC were entered as covariates. Clinical measures of upper-limb impairment such as the CMSA were not included as covariates to avoid multicollinearity and because they were less correlated with motor skill performance than MVC. We used a restricted (or residual) maximum likelihood, a common method used for fitting LMMs, assuming that missing data is missing at random. Participants’ random intercepts and slopes were included in the models.

LMMs with the same structure were also used to assess differences between cortical and subcortical groups for CSE. In this case, each specific CSE measure was the dependent variable, and group was the fixed effect. Stroke severity (NIHSS), age, and time since stroke (days) were the covariates entered in the models. In this case, MVC was not included as covariate in the model to reduce redundancy. All LMMs were corrected using Tukey HSD adjustment for multiplicity.

Multivariate linear regression models were employed to investigate associations between CSE and motor performance for cortical and subcortical groups, including the same covariates as in the motor skill performance LMMs. Separate models analysed CSE from ipsilesional and contralesional hemispheres. To test whether the associations between CSE measures and motor performance differed by lesion location, the interaction between lesion location (cortical versus subcortical) and each CSE measure was included in the regression models. Multicollinearity between covariates was assessed with the variance inflation factor (VIF), with a threshold of >5 indicating excessive multicollinearity. Linear model assumptions were checked for residual normality. Influential observations were identified using quantile range outliers (tail quantile = 0.1, Q = 3) for each variable, and leverage plots and Cook’s Distance (score >1) in the regression model. We did not apply a false discovery rate adjustment because each regression model investigated associations for different CSE measures. Accordingly, all reported *P* values are uncorrected for multiple comparisons.

The exploratory analysis compared individuals with cortical lesions to those with subcortical lesions affecting only CST-associated regions or subcortical regions without direct CST involvement, examining their impact on motor skill performance, CSE, and their association. As previously stated, besides allowing us to investigate the role of the CST, this exploratory analysis was also used to confirm that our lesion location subgroup categorization (i.e. cortical versus subcortical) was accurate. Furthermore, to confirm the results of the primary regression analyses, we also conducted a sensitivity analysis removing participants with cortical-subcortical lesions and thus including only individuals with pure cortical lesions. All analyses were performed with two-tailed test using JMP (version 17 Pro) from SAS. The alpha level was set at <0.05.

## Results

### Sample characteristics

After removing from the analysis 13 individuals with lesions affecting the cerebellum and/or brainstem, a total of 103 participants were classified based on lesion location as cortical (*n* = 34) or subcortical (*n* = 69) (Table 1). Within the subcortical group, 26 participants had lesions affecting the CST and 43 participants lesions not affecting this structure. Results are presented using a stepwise approach, beginning with the primary analysis comparing cortical and subcortical lesions, followed by the exploratory analysis examining CST involvement across three groups (cortical, subcortical CST-unaffected, and subcortical CST-affected). Time since stroke (days) was longer in the subcortical groups, although this difference became non-significant when groups were stratified as subacute (<6 months) and chronic (>6 months) categories. In individuals with subcortical lesions, measures of upper-limb motor impairment (CMSA_arm,_ CMSA_hand_), hand strength (MVC_affected_) and function (BBT_affected_) of the most affected hand exhibited larger deficits (Table 1).

**Table 1:** Demographic and clinical data for cortical and subcortical groups

	Cortical	Subcortical	*P* value
*N*	34	69	
Age (y)	64.79 (11.61)	65.27 (9.51)	0.823
Sex (F/M)	7/27	25/44	0.107
Subacute/Chronic	28/6	41/28	0.020*
Days since stroke	135.94 (199.85)	315.13 (429.86)	0.025*
Subacute	63.64 (22.85)	65.29 (23.11)	0.292
Chronic	473.33 (308.08)	680.96 (480.64)	0.321
Type of stroke (I/H)	33/1	60/9	0.103
Lesion location	Frontal = 29.6%	Corona radiata = 17.4%	
	Parietal = 14.7%	Basal ganglia = 21.8%	
	Occipital = 5.8%	Internal capsule = 18.7%	
	Multiple cortical regions = 14.7%	Thalamus = 10.9%	
	Cortico-Subcortical = 35.2%	Multiple subcortical regions = 31.2%	
NIHSS	1.79 (1.73)	1.97 (2.09)	0.671
MoCA	23.75 (4.95)	23.56 (4.98)	0.857
CMSA_arm_	6.54 (1.09)	5.41 (1.80)	0.0004*
CMSA_hand_	6.42 (0.87)	5.32 (1.88)	0.0061*
MVC_affected_	0.79 (0.25)	0.63 (0.31)	0.0070*
MVC_unaffected_	0.86 (0.21)	0.83 (0.29)	0.641
BBT_affected_	49.57 (11.44)	41.40 (15.33)	0.0100*
BBT_unaffected_	52.12 (7.74)	49.72 (9.67)	0.221

BBT, Box and Block Test; CMSA, Chedoke-McMaster Stroke Assessment; M, Male; MoCA, Montreal Cognitive Assessment; MVC, Maximal voluntary contraction; NIHSS, National Institutes of Health Stroke Scale; F, Female. Values are presented as means and standard deviations (SD). *P < 0.05.

The exploratory analyses (Supplementary Table 1) indicated no significant differences among the three lesion groups (cortical, subcortical with CST affected, and subcortical with CST unaffected) in age (F_(2,100)_ = 0.09; *P* = 0.906), sex (X^2^_(2,103)_ = 3.262; *P* = 0.196), time since stroke (F_(2,100)_  _=_ 2.75; *P* = 0.063), stroke severity (F_(2,100)_ = 0.20; *P* = 0.819), and cognitive status (F_(2,100)_ = 3.05; *P* = 0.052). However, participants with subcortical lesions affecting the CST showed significantly worse CMSA_arm_ (F_(2,94)_ = 9.50; *P* = 0.0002), CMSA_hand_ (F_(2,95)_ = 7.89; *P* = 0.0007), MVC_affected_ (F_(2,93)_ = 5.42; *P* = 0.0060) and BBT_affected_ (F_(2,96)_ = 4.06; *P* = 0.0203).

### Motor skill performance

**Figure 3:**
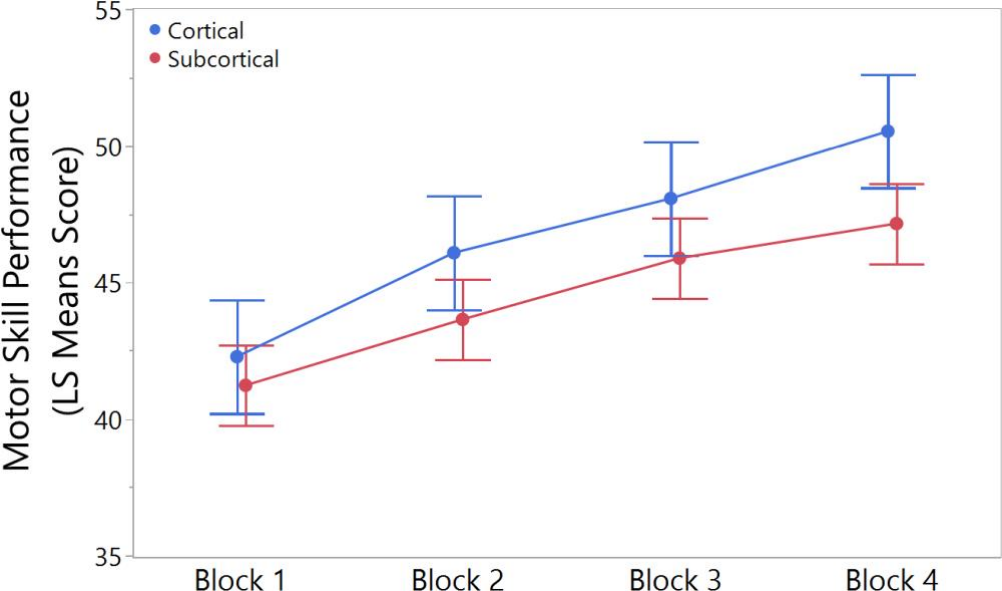
**Group scores in motor skill performance**. There were no significant differences between the cortical and subcortical groups in motor skill performance at baseline (Block 1) (t = 0.40; *P* = 0.999) and improvements over motor practice were not significantly different between groups (F(3,169.9) = 1.27, *P* = 0.287. Data is presented as least squares mean estimates with SEM. Statistical analysis was performed using repeated linear mixed models (LMMs) with participant as the experimental unit (*n* = 103; 69 subcortical, 34 cortical strokes). LMMs were adjusted for stroke severity, age, handgrip maximal voluntary contraction and time since stroke.

Two trials of the visuomotor adaptation task are shown in Fig. 2 as example. There were no significant differences between the cortical and subcortical groups in motor skill performance at baseline (Block 1) (t = 0.40; *P* = 0.999) and improvements over motor practice were not significantly different between groups (F_(3,169.9)_ = 1.27, *P* = 0.287) (Fig. 3) even when LMMs were not adjusted with covariates (Supplementary Tables 2.1–2.2). Similarly, in the exploratory sensitivity analyses, both adjusted (F_(6,193.3)_ = 0.70, *P* = 0.652) and unadjusted (F_(6,212.7)_ = 0.82, *P* = 0.552) LMMs revealed no significant differences among the three lesion groups in improvements in motor skill performance (Supplementary Tables 2.3–2.4).

#### Cortico-spinal excitability

A total of 0.04% of TMS trials were removed due to excessive background muscle activity or other unexpected experimental events. CSE measures were obtained from all participants except 5, whose MEPs could not be elicited on either hemisphere. For 10 participants with no ipsilesional response, only contralesional hemisphere data were used. Motor outcome data, including motor skill performance from individuals without MEPs in one or both hemispheres were still included in the behavioural analyses. Only active MEP differed significantly between cortical and subcortical groups. Differences in the rest of ipsilesional CSE measures were not statistically significant. On average, the ICF and SICI protocols demonstrated facilitation (>100%) and inhibition (<100%), respectively (Table 2). However, exploratory one sample t-tests revealed that some participants with cortical lesions did not show significant inhibition in the ipsilesional hemisphere. Details are provided in Supplementary Table 3.

**Table 2:** Cortico-spinal excitability (CSE) measures in ipsilesional and contralesional hemispheres

	Cortical	Subcortical	*P* value
Ipsilesional			
RMT (%)	48.84 (2.19)	54.16 (1.59)	0.056
Resting MEP (mV)	0.51 (0.07)	0.38 (0.05)	0.126
Active MEP (mV)	1.74 (0.15)	1.33 (0.11)	0.031*
CSP (ms)	0.19 (0.01)	0.19 (0.01)	0.982
ICF (%)	171.51 (20.27)	191.29 (15.42)	0.439
SICI (%)	93.18 (11.27)	74.45 (8.18)	0.195
Contralesional			
RMT (%)	51.74 (1.91)	51.38 (1.32)	0.878
Resting MEP (mV)	0.47 (0.04)	0.41 (0.03)	0.303
Active MEP (mV)	1.71 (0.14)	1.76 (0.10)	0.772
CSP (ms)	0.18 (0.01)	0.17 (0.01)	0.255
ICF (%)	173.35 (23.46)	190.95 (16.99)	0.569
SICI (%)	54.93 (16.38)	83.77 (11.89)	0.141

CSP, cortical silent period; ICF, intracortical facilitation; MEP, motor evoked potential; RMT, resting motor threshold; SICI short-intracortical inhibition. Values are means estimates and standard errors of the mean (SEM). LMMs were adjusted for stroke severity, age and time since stroke.

^*^ *P* < 0.05.

The results of the exploratory analyses revealed that participants with lesions involving the CST tended to show less excitability in the ipsilesional hemisphere, as indicated by significantly smaller resting (F_(2,82)_ = 4.92, *P* = 0.0091) and active (F_(2,82)_ = 8.29, *P* = 0.0005) MEP amplitudes (Supplementary Table 4). No significant differences among the three groups were found in the other ipsilesional or contralesional CSE measures. Details on the LMMs comparing CSE among the three lesion groups are provided in Supplementary Table 5.

### Associations between excitability and motor skill performance

**Figure 4:**
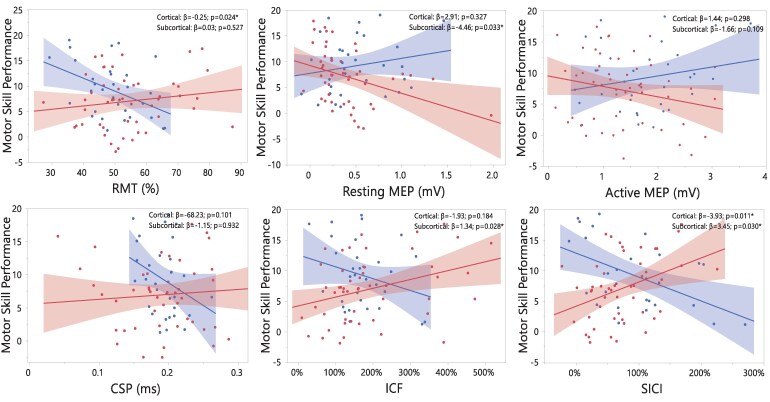
**Partial regression plots showing the associations between each CSE measure in the ipsilesional hemisphere and motor skill performance in cortical and subcortical lesions.** Cortical and subcortical groups are depicted in blue and red, respectively. Statistical analysis was performed using multivariate linear regression models with participant as the experimental unit (*n* = 103; 69 subcortical, 34 cortical strokes). Models were adjusted for stroke severity, age, handgrip maximal voluntary contraction, and time since stroke. CSP, cortical silent period; ICF, intracortical facilitation; MEP, motor evoked potential; RMT, resting motor threshold; SICI, short-intracortical inhibition. Note that in ICF and SICI conditioned MEP amplitude is normalized to the unconditioned resting MEP amplitude, with values >100% indicating ICF and values <100% indicating SICI. *P < 0.05.

**Figure 5:**
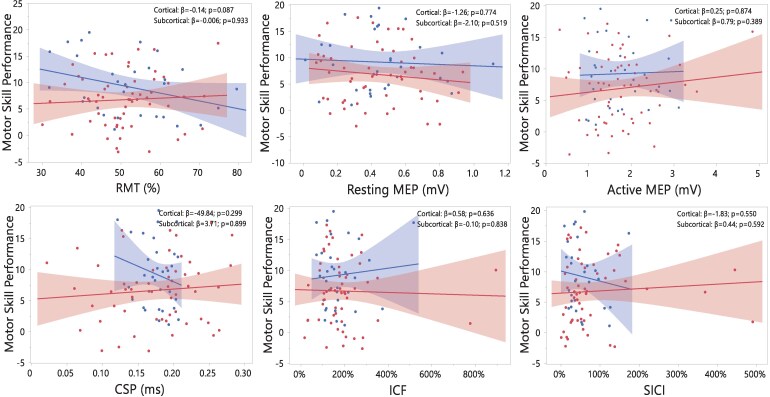
**Partial regression plots showing the associations between each CSE measure in the contralesional hemisphere and motor skill performance in cortical and subcortical lesions.** Cortical and subcortical groups are depicted in blue and red, respectively. Statistical analysis was performed using multivariate linear regression models with participant as the experimental unit (*n* = 103; 69 subcortical, 34 cortical strokes). Models were adjusted for stroke severity, age, handgrip maximal voluntary contraction, and time since stroke. CSP, cortical silent period; ICF, intracortical facilitation; MEP, motor evoked potential; RMT, resting motor threshold; SICI, short-intracortical inhibition. Note that in ICF and SICI conditioned MEP amplitude is normalized to the unconditioned resting MEP amplitude, with values >100% indicating ICF and values <100% indicating SICI. *P < 0.05.

Twelve influential observations from five participants were identified and removed from the regression analyses (Ipsilesional: ICF = 2, SICI = 2, CSP = 2; Contralesional: ICF = 2, SICI = 2, CSP = 2). Importantly, removing these influential observations had no effect on the regression results. Divergent associations between several CSE measures and motor skill performance were observed in cortical and subcortical groups in the ipsilesional hemisphere (Fig. 4). In individuals with cortical lesions, motor skill performance was associated with lower RMT (β = −0.25, 95% CI [−0.47, −0.03]; *P* = 0.024) and increased SICI (β = −3.93, 95% CI [−6.89, −0.98]; *P* = 0.011) in the ipsilesional hemisphere. In contrast, in individuals with subcortical lesions, motor skill performance was associated with smaller resting MEP amplitude (β = −4.46, 95% CI [−8.54, −0.38]; *P* = 0.033), increased ICF (β = 1.34, 95% CI [0.15,2.54]; *P* = 0.028), and reduced SICI (β = 3.45, 95% CI [0.34,6.56]; *P* = 0.030) in the ipsilesional hemisphere (Supplementary Table 6). Group differences in the strength of associations between CSE measures and motor performance were confirmed by significant lesion location × CSE interactions for RMT (*P* = 0.013), resting MEP amplitude (*P* = 0.039), ICF (*P* = 0.041), and SICI (*P* = 0.001) (Supplementary Table 8). In the contralesional hemisphere neither the cortical nor subcortical group exhibited any significant association (Fig. 5) (Supplementary Table 7).

The exploratory analyses revealed that associations between CSE and motor skill performance within the subcortical group were driven by observations from individuals with CST lesions. This is, associations with SICI (β = 10.31, 95% CI [2.07,18.54]; *P* = 0.019) and ICF (β = 2.64, 95% CI [0.34,4.94]; *P* = 0.027) remained significant only when the data of the subcortical group with CST lesions were analysed. No significant associations were found in the contralesional hemisphere. For the sensitivity analysis used to confirm the results of the primary regression analysis 13 participants with cortical-subcortical lesions were removed from the cortical group. This analysis confirmed the direction of the associations observed in the primary analysis for the cortical group but, while the association with increased SICI remained significant (β = −5.68, 95% CI [−10.03, −1.34]; *P* = 0.015), the association with lower RMT became non-significant (β = −0.28, 95% CI [−0.63, 0.08]; *P* = 0.117). Details of all regression models are provided in Supplementary Tables 6–12.

## Discussion

The CST is a collection of axons stemming from multiple territories in the sensorimotor cortex, primarily M1, but also from premotor areas and supplementary motor areas, descending in a funnel-like manner to converge subcortically into the corona radiata, internal capsule and peduncles.^34^ Damage to the CST is the common denominator for post-stroke hemiparesis and can lead to significant deficits in upper-limb function, with studies identifying its degree of lesion as the best predictor of precision grip function.^2^ Taken together, our findings support the view that lesions in subcortical brain areas where descending motor pathways are densely compacted are more likely to produce upper-limb impairments than more superficial cortical lesions and that subcortical lesions affecting the CST tend to augment these impairments.^8^

We assessed motor skill performance using a handgrip short-term visuomotor adaptation task that mimics essential activities of daily living such as grasping and manipulating objects.^22^ The performance of these tasks, which are correlated with upper-limb function, is thought to rely on the integrity of the CST.^2,35^ Considering the influence that descending cortico-spinal projections and subcortical brain regions have on motor skill acquisition,^36^ upper-limb reaching and hand manipulation tasks, one would expect worse motor skill performance in the subcortical group, especially if the lesion affects the CST.^37^ However, while performance tended to be lower in individuals with subcortical lesions, improvement rates did not differ between groups. This result aligns with previous studies suggesting a relatively well-preserved capacity to improve motor performance post-stroke.^38,39^

This study did not have an age-matched control group to establish a direct comparison but a previous study involving 26 neurotypical older adults (70.60 [8.07] years) that used a pinching variant of the same visuomotor adaptation task showed a mean average improvement of 11.14(6.22) points during practice^35^ while in the present study, participants improved only 7.7(6.17) points. This difference, however, could be even greater if task difficulty is considered. This is, in the study involving neurotypical individuals, the task was more challenging because the distance among targets was increased, and thus participants needed to apply more force variations to reach the different targets. Hence, despite people with stroke having a relatively well-preserved capacity to improve motor performance in this visuomotor adaptation task, it seems that healthy individuals, even of older age, can improve motor performance at a faster rate.

Differences between groups in CSE emerged only in the ipsilesional hemisphere, with the subcortical group showing smaller MEP amplitudes assessed during the performance of an active contraction (Table 2). It is possible that this reduced CSE becomes only apparent during muscle contraction due to spinal facilitation mechanisms, which have shown to increase MEP amplitudes when force is produced.^31^ Additionally, despite using significantly higher TMS intensities, the subcortical group also showed a tendency to have higher RMTs and produce smaller resting MEP amplitudes, suggesting reduced CSE. However, differences between groups in these two CSE measures did not reach statistical significance. Exploratory analyses revealed that reductions in active and resting excitability in the ipsilesional hemisphere were exacerbated in those individuals with subcortical lesions affecting the CST (Supplementary Table 4). Taken together, these findings align with most, but not all,^40^ previous studies showing that subcortical lesions lead to a greater overall reduction of excitability in the ipsilesional hemisphere.^16^ Furthermore, this reduction appears to be more substantial when descending motor pathways in subcortical areas are damaged,^41^ supporting a link between structural and functional CST damage.^42^ Studies analysing other features of the MEP such as its shape (e.g. monophasic or polyphasic) would provide additional insights regarding how lesion location changes different aspects of CSE.^43^

At face value, the lack of significant differences between groups in CSP, ICF and SICI observed in this study may seem at odds with a study of smaller size (*n* = 40) but with a more precise characterization of the lesion location that showed both greater reductions in SICI (i.e. disinhibition) in individuals with cortical lesions, and longer prolongations of the CSP in individuals with subcortical lesions.^16^ We found that in individuals with cortical lesions, inhibition was more difficult to elicit in the ipsilesional than in the contralesional hemisphere (Table 2). Furthermore, exploratory one sample t-tests revealed that some participants with cortical lesions did not show significant inhibition in the ipsilesional hemisphere, a finding that has been reported previously.^44^ The study with a more precise characterization of the lesion location, however, only included acute and very early subacute patients (<14 days), who typically exhibit greater reductions in SICI and increases in CSP than individuals in later stages of recovery.^13^ Our analyses included time since stroke as covariate to factorize the impact of stroke recovery stage, which had little influence in the LMM (Supplementary Tables 3 and 5). More importantly, comparisons between cortical and subcortical groups in the study mentioned previously^16^ were made using the interhemispheric ratios (ipsilesional/contralesional) of these CSE measures. Therefore, these results cannot be directly compared because interhemispheric ratios were not assessed in our study. Overall, our results do not support the view that lesion location has a significant influence in GABA-mediated inhibition.^45^

We found striking differences in the associations between CSE measures obtained from the ipsilesional hemisphere and motor skill performance in individuals with cortical and subcortical lesions (Fig. 4). Importantly, these differences were not influenced by time since stroke, a variable that like in the LMMs used to study differences between groups in motor skill performance and CSE, was controlled in all regression models. In individuals with cortical lesions, more excitability in the ipsilesional hemisphere, expressed as reduced RMT, was significantly associated with improvements in motor skill performance. This association is consistent with the much-debated hypothesis that CSE levels can, sometimes, predict motor performance and motor skill learning on a group level.^46,47^ Indeed, while prior studies in neurotypical individuals usually do not examine associations between CSE measures at rest and motor skill performance capabilities, similar neurophysiological responses such as increased MEP amplitudes are reported following different forms of motor skill practice.^35,48,49^ In contrast, we found that in individuals with subcortical lesions, it was lower CSE, expressed as smaller resting MEP amplitudes, which was associated with better motor performance. This association was not expected because it is precisely in those individuals with more subcortical CST damage where smaller MEPs should be associated with poorer motor performance.^46^ The exact reason for this negative association is still to be elucidated, but it could be related to the recruitment of alternative networks during motor practice.

Indeed, in individuals with subcortical lesions, motor skill learning has been associated with increased activity and connectivity in frontal brain regions alongside reduced activity in motor areas, implying a reliance on compensatory cortical changes to preserve learning capacity.^50^ It is therefore possible that, in these individuals, reduced CSE could reflect, paradoxically, an increased recruitment of alternative cortical circuits that support motor performance when CST connections are disrupted.^11^ Redistribution of synaptic strength and neural excitability within a dynamic range might be crucial for maintaining stability amongst motor networks.^51^ Neuroimaging studies have demonstrated that subcortical lesions disrupting the CST can trigger increased activity in secondary motor regions and the recruitment of residual pathways as a compensatory mechanism to preserve cortico-spinal outputs.^11^

We also found marked differences between cortical and subcortical groups in the associations between motor performance and CSE measures of facilitation (ICF) and inhibition (SICI) in the ipsilesional hemisphere (Fig. 4). The primary motor cortex (M1) and its descending cortico-spinal projections are under GABAergic influence, with inhibitory neurons suppressing any excitatory drive emerging from the neighbouring intracortical representations and thus regulating neuronal action potential firing.^52^ Following ischaemia or hypoxic damage, phasic GABA-related signalling is dramatically reduced, unmasking normally suppressed connections and shifting the excitatory balance towards facilitation.^53^ Such changes in facilitatory-inhibitory balance have been shown to be critical for inducing neuroplastic structural changes that support both motor recovery and skill learning.^5,54^ In animal models with motor pathway lesions, reducing inhibition can lead to sustained motor recovery improvements.^55^ Similarly, in individuals post-stroke, reduced SICI has been observed across the recovery continuum and associated with the degree of recovery,^24^ something that has also been observed in neurotypical individuals following motor practice and linked with motor skill learning.^49,56^ Multiple studies have attempted to investigate the functional implications of this excitatory-inhibitory balance in post-stroke survivors and the implication for motor recovery, but findings remain still inconclusive.^57^

Our study provides novel insights into the functional role of this excitatory-inhibitory balance by revealing the existence of two distinct intracortical excitability patterns underlying motor skill performance depending on lesion location. In individuals with cortical lesions, better performance rates were associated with greater inhibitory activity, suggesting neurophysiological patterns similar to neurotypical individuals when lesions spare cortico-spinal pathways.^48^ Conversely, in subcortical lesions, where descending cortico-spinal fibres tend to be more severely compromised, a shift in the facilitation-inhibition balance might be needed in order to preserve motor skill performance capacity, with reduced intracortical inhibition unmasking latent excitatory glutamatergic connections.^58^ Our exploratory analyses confirmed that both ICF and SICI associations in the subcortical group were primarily driven by damage in the CST because, only in individuals with lesions in this pathway, these associations remained significant (Supplementary Table 9). These findings reinforce previous evidence in support of the critical role that GABAergic and glutamatergic balance has in brain repair and recovery processes after stroke.^15^

Given the neuroanatomy of the CST, the fact that no statistically significant associations with motor skill performance were observed for any of the CSE measures obtained from the contralesional hemisphere was not completely unexpected (Fig. 5). This finding aligns with previous experimental and clinical studies indicating that improvements in upper-limb recovery derive from changes in activity taking place primarily in peri-infarct neurons in the ipsilesional hemisphere.^59^ Associations between CSE and the contralesional hemisphere may become significant only in individuals with more severe subcortical lesions who present a substantial or complete disruption of cortico-spinal connections.^17^ Since our study included mostly individuals with relatively mild levels of upper-limb motor impairment (Table 1), a significant disruption of the CST projections possibly occurred only in a very small proportion of our participants.

The mechanistic insights discovered in this study could be relevant for clinical practice. Modifying CSE by either inhibiting or facilitating intracortical networks is the backbone of most non-invasive brain stimulation (NIBS) treatments aimed at improving upper-limb motor recovery after stroke. Despite multiple efforts, there is currently no consistent evidence that these interventions benefit upper-limb recovery, in part because its treatment response can be influenced by numerous factors including lesion location.^60^ Consensus-based recommendations for NIBS in stroke rehabilitation highlight the need for increased understanding regarding response phenotypes and neural mechanisms in order to improve individualization of treatment protocols.^61^ Our results provide novel neurophysiological associations between CSE and motor skill performance that could be used to identify more accurate therapeutical targets to promote recovery.

## Limitations

The most important limitation of this study is the lack of raw structural neuroimaging data access to characterize more precisely the stroke lesion and the extent of the CST damage. To mitigate this limitation, lesion classification was performed by experienced neurologists and neuroimaging experts, with collective review in cases of uncertainty. Furthermore, the results of our exploratory analysis, dividing patients in three groups based on the stroke location (cortical, and subcortical with and without CST damage), provide support for our lesion classification. This analysis confirmed, as one would expect, that patients with subcortical lesions affecting the CST had more upper-limb impairment, less handgrip force, and less resting and active excitability than patients with cortical lesions or subcortical lesions without CST. Since these three groups were similar in terms of age, sex and time since stroke (Supplementary Table 1), differences are most likely explained by lesion location.

In any event, we acknowledge that obtaining high resolution MRI scans once the perilesional oedema has remitted and thus when the quantification of neurological damage is more accurate, would have certainly improved the precision in which lesions were located. Similarly, obtaining more specific data on the lesion load affecting the CST (e.g. anisotropy, diffusion-weighted imaging) along with information contributing to variability in CSE and motor behaviour such as other surviving neural pathways, primary and secondary motor areas, and white matter hyperintensities, would have strengthened the interpretation of our results.^62,63^ Admitting the fact that brain lesions do not fit into a simple binary classification, our study allowed us to address a specific mechanistic question, demonstrating the important role of lesion location in mediating the neurophysiological properties underlying motor skill performance post-stroke.

Methodology pertaining to the application of TMS may have also influenced our findings and should be taken into consideration. Firstly, we cannot rule out that our intertrial interval (5 s) may have not influenced CSE responses.^64,65^ However, given the length of each TMS assessment session (∼1.5 h) and the large number of measures collected from each hemisphere, increasing the time between trials more than 5 s would have increased the duration of the assessments, which could have had detrimental effects (e.g. increase patients’ fatigue or affect attention). Notably, participants also underwent the same TMS protocols with identical intertrial interval so, although interindividual differences are possible,^64^ any potential neuromodulatory effect should in principle affect all patients similarly.

Thirdly, to assess SICI, in some participants we employed an ISI (2.5 ms) that we have used in previous stroke studies^22^ and that has shown to effectively suppress the MEP response.^66^ Some studies suggest that ISIs of 2 ms are more appropriate for SICI because longer ISIs may be influenced by facilitatory mechanisms.^67^ However, when we compared the observations derived from ISIs of 2.5 and 2 ms to assess SICI used in this study we found no significant differences. This was confirmed by equivalence tests showing that both ISIs were equivalent in eliciting inhibition. While we cannot rule out completely the potential influence of facilitatory mechanisms in SICI, on a group level, the ISIs used in our study were effective at reducing the conditioned MEP and thus produced inhibition (Table 2). It is also important to note that SICI is a complex measure and that, compared to healthy individuals,^67^ people with stroke may respond differently to different ISIs ranges.^68^

Another limitation concerns the level of motor disability of the participants and how this limited the generalizability of our results to individuals with more severe upper-limb impairments. It is possible that people with more severe lesions could show different patterns in the associations between CSE and motor skill performance.^17^ Nevertheless, about 65% of stroke survivors exhibit coordination and control deficits in the affected hand, even in cases with mild or no motor impairment.^2^ Including participants with relatively moderate levels of upper-limb motor impairment allowed us to assess motor skill performance using a highly functional motor task and to obtain complete CSE measures from most participants. Obtaining TMS data in very impaired patients whose MEP responses from the ipsilesional hemisphere cannot be easily elicited is challenging.^69^ Future studies should validate to what extent our results can be extrapolated to more impaired individuals.

Another limitation refers to the lack of a delayed retention test of the visuomotor adaptation task to investigate the capacity to retain the gains in motor skill acquisition obtained during practice. Motor skill learning is a complex multistage process that requires encoding sensorimotor information during motor practice but also the consolidation of such information as procedural memory.^70^ To evaluate motor skill learning and distinguish it from potential transient improvements in motor skill performance occurring during motor practice a delayed retention test of the practiced motor task would have been needed.^71^ Future studies investigating whether associations between CSE measures and motor skill performance remain significant when retention is assessed and the influence of lesion location are warranted.^72^

## Conclusion

Understanding the basic neurophysiological mechanisms underlying distinct phases of motor skill learning after stroke has the potential to impact recovery and rehabilitation significantly.^18^ However, the ubiquitous heterogeneity in human stroke studies limits the identification of unique neural patterns that could improve the ability to predict long-term outcomes and responses to treatments.^73^ This study sheds light on this issue by showing the influential role of lesion location in upper-limb motor skill performance and its underlying neurophysiological mechanisms. Contrary to what we observed in motor impairment and function, motor skill performance was relatively well preserved in individuals with subcortical lesions, who, compared with individuals with cortical lesions, tended to show lower levels of excitability in the ipsilesional hemisphere. Furthermore, while better motor skill performance was associated with neurotypical excitatory patterns in cortical strokes, lower excitability, intracortical inhibition and higher facilitation correlated with larger performance rates in subcortical lesions. These results indicate that motor skill performance may rely on distinct cortico-spinal circuits depending on lesion location after stroke and suggest that alternative networks may play an important role in preserving acquisition in those lesions affecting descending cortico-spinal pathways. We hope that these findings will offer valuable insights to identify more accurate therapeutical targets for the design of treatment approaches aimed at promoting recovery following stroke.

## Supplementary Material

fcaf430_Supplementary_Data

## Data Availability

Data is available upon reasonable request.

## References

[1] Kwakkel G, Kollen BJ, Van Der Grond J, Prevo AJH. Probability of regaining dexterity in the flaccid upper limb. Stroke. 2003;34(9):2181–2186. 12907818 10.1161/01.STR.0000087172.16305.CD

[2] Pennati GV, Plantin J, Carment L, et al Recovery and prediction of dynamic precision grip force control after stroke. Stroke. 2020;51(3):944–951. 31906829 10.1161/STROKEAHA.119.026205

[3] Kitago T, Krakauer JW. Motor learning principles for neurorehabilitation. Handb Clin Neurol. 2013;110:93–103. 23312633 10.1016/B978-0-444-52901-5.00008-3

[4] Metz GA, Antonow-Schlorke I, Witte OW. Motor improvements after focal cortical ischemia in adult rats are mediated by compensatory mechanisms. Behav Brain Res. 2005;162(1):71–82. 15922067 10.1016/j.bbr.2005.03.002

[5] Joy MT, Carmichael ST. Encouraging an excitable brain state: Mechanisms of brain repair in stroke. Nat Rev Neurosci. 2021;22(1):38–53. 33184469 10.1038/s41583-020-00396-7 PMC10625167

[6] Ward NS . Restoring brain function after stroke—Bridging the gap between animals and humans. Nat Rev Neurol. 2017;13(4):244–255. 28303914 10.1038/nrneurol.2017.34

[7] Cramer SC . Stratifying patients with stroke in trials that target brain repair. Stroke. 2010;41(10_suppl_1):S114–S116. 20876483 10.1161/STROKEAHA.110.595165

[8] Shelton FN, Reding MJ. Effect of lesion location on upper limb motor recovery after stroke. Stroke. 2001;32(1):107–112. 11136923 10.1161/01.str.32.1.107

[9] Stinear CM, Byblow WD, Ackerley SJ, Smith M-C, Borges VM, Barber PA. PREP2: A biomarker-based algorithm for predicting upper limb function after stroke. Ann Clin Transl Neurol. 2017;4(11):811–820. 29159193 10.1002/acn3.488 PMC5682112

[10] Luft AR, Waller S, Forrester L, et al Lesion location alters brain activation in chronically impaired stroke survivors. NeuroImage. 2004;21(3):924–935. 15006659 10.1016/j.neuroimage.2003.10.026

[11] Ward NS, Newton JM, Swayne OB, et al Motor system activation after subcortical stroke depends on corticospinal system integrity. Brain. 2006;129(Pt 3):809–819. 16421171 10.1093/brain/awl002 PMC3717515

[12] Boyd LA, Hayward KS, Ward NS, et al Biomarkers of stroke recovery: Consensus-based core recommendations from the stroke recovery and rehabilitation roundtable. Int J Stroke. 2017;12(5):480–493. 28697711 10.1177/1747493017714176 PMC6791523

[13] McDonnell MN, Stinear CM. TMS measures of motor cortex function after stroke: A meta-analysis. Brain Stimul. 2017;10(4):721–734. 28385535 10.1016/j.brs.2017.03.008

[14] Ziemann U, Reis J, Schwenkreis P, et al TMS and drugs revisited 2014. Clin Neurophysiol. 2015;126(10):1847–1868. 25534482 10.1016/j.clinph.2014.08.028

[15] Carmichael ST . Brain excitability in stroke: The yin and yang of stroke progression. Arch Neurol. 2012;69(2):161–167. 21987395 10.1001/archneurol.2011.1175 PMC4698890

[16] Liepert J, Restemeyer C, Kucinski T, Zittel S, Weiller C. Motor strokes. Stroke. 2005;36(12):2648–2648. 16269647 10.1161/01.STR.0000189629.10603.02

[17] Thickbroom GW, Cortes M, Rykman A, et al Stroke subtype and motor impairment influence contralesional excitability. Neurology. 2015;85(6):517–520. 26187228 10.1212/WNL.0000000000001828 PMC4540249

[18] Krakauer JW . Motor learning: Its relevance to stroke recovery and neurorehabilitation. Curr Opin Neurol. 2006;19:84–90. 16415682 10.1097/01.wco.0000200544.29915.cc

[19] Riga A, Gathy E, Ghinet M, et al Evidence of motor skill learning in acute stroke patients without lesions to the thalamus and internal capsule. Stroke. 2022;53(7):2361–2368. 35311345 10.1161/STROKEAHA.121.035494 PMC9232242

[20] Boyd LA, Winstein CJ. Providing explicit information disrupts implicit motor learning after basal ganglia stroke. Learn Mem. 2004;11(4):388–396. 15286181 10.1101/lm.80104 PMC498316

[21] Von Elm E, Altman DG, Egger M, Pocock SJ, Gøtzsche PC, Vandenbroucke JP. The strengthening the reporting of observational studies in epidemiology (STROBE) statement: Guidelines for reporting observational studies. J Clin Epidemiol. 2008;61(4):344–349. 18313558 10.1016/j.jclinepi.2007.11.008

[22] Nepveu J-F, Thiel A, Tang A, et al A single bout of high-intensity interval training improves motor skill retention in individuals with stroke. Neurorehabil Neural Repair. 2017;31(8):726–735. 28691645 10.1177/1545968317718269

[23] Rossi S, Hallett M, Rossini PM, Pascual-Leone A. Safety, ethical considerations, and application guidelines for the use of transcranial magnetic stimulation in clinical practice and research. Clin Neurophysiol. 2009;120(12):2008–2039. 19833552 10.1016/j.clinph.2009.08.016 PMC3260536

[24] Huynh W, Vucic S, Krishnan AV, Lin CS, Kiernan MC. Exploring the evolution of cortical excitability following acute stroke. Research support. Non-U.S. Gov't. Neurorehabil Neural Repair. 2016;30(3):244–257. 26150146 10.1177/1545968315593804

[25] Park CH, Kou N, Ward NS. The contribution of lesion location to upper limb deficit after stroke. J Neurol Neurosurg Psychiatry. 2016;87(12):1283–1286. 27451352 10.1136/jnnp-2015-312738 PMC5136717

[26] Lyden P, Lu M, Jackson C, et al Underlying structure of the national institutes of health stroke scale: Results of a factor analysis. NINDS tPA stroke trial investigators. Stroke. 1999;30(11):2347–2354. 10548669 10.1161/01.str.30.11.2347

[27] Nasreddine ZS, Phillips NA, Bedirian V, et al The Montreal cognitive assessment, MoCA: A brief screening tool for mild cognitive impairment. J Am Geriatr Soc. 2005;53(4):695–699. 15817019 10.1111/j.1532-5415.2005.53221.x

[28] Gowland C, Stratford P, Ward M, et al Measuring physical impairment and disability with the Chedoke-McMaster stroke assessment. Stroke. 1993;24(1):58–63. 8418551 10.1161/01.str.24.1.58

[29] Lin K-C, Chuang L-L, Wu C-Y, Hsieh Y-W, Chang W-Y. Responsiveness and validity of three dexterous function measures in stroke rehabilitation. J Rehabil Res Dev. 2010;47(6):563. 20848369 10.1682/jrrd.2009.09.0155

[30] Kleim JA, Kleim ED, Cramer SC. Systematic assessment of training-induced changes in corticospinal output to hand using frameless stereotaxic transcranial magnetic stimulation. Nat Protoc. 2007;2(7):1675–1684. 17641632 10.1038/nprot.2007.206

[31] Rossini PM, Burke D, Chen R, et al Non-invasive electrical and magnetic stimulation of the brain, spinal cord, roots and peripheral nerves: Basic principles and procedures for routine clinical and research application. An updated report from an I.F.C.N. Committee. Clin Neurophysiol. 2015;126(6):1071–1107. 25797650 10.1016/j.clinph.2015.02.001 PMC6350257

[32] Tunovic S, Press DZ, Robertson EM. A physiological signal that prevents motor skill improvements during consolidation. J Neurosci. 2014;34(15):5302–5310. 24719108 10.1523/JNEUROSCI.3497-13.2014 PMC3983806

[33] Saisanen L, Pirinen E, Teitti S, et al Factors influencing cortical silent period: Optimized stimulus location, intensity and muscle contraction. J Neurosci Methods. 2008;169(1):231–238. 18243329 10.1016/j.jneumeth.2007.12.005

[34] He S, Dum R, Strick P. Topographic organization of corticospinal projections from the frontal lobe: Motor areas on the lateral surface of the hemisphere. J Neurosci. 1993;13(3):952–980. 7680069 10.1523/JNEUROSCI.13-03-00952.1993 PMC6576595

[35] Centeno C, Medeiros D, Beck MM, et al The effects of aging on cortico-spinal excitability and motor memory consolidation. Neurobiol Aging. 2018;70:254–264. 30053741 10.1016/j.neurobiolaging.2018.06.035

[36] Doyon J, Bellec P, Amsel R, et al Contributions of the basal ganglia and functionally related brain structures to motor learning. Behav Brain Res. 2009;199(1):61–75. 19061920 10.1016/j.bbr.2008.11.012

[37] Lemon RN, Griffiths J. Comparing the function of the corticospinal system in different species: Organizational differences for motor specialization? Muscle Nerve. 2005;32(3):261–279. 15806550 10.1002/mus.20333

[38] Winstein CJ, Merians AS, Sullivan KJ. Motor learning after unilateral brain damage. Neuropsychologia. 1999;37:975–987. 10426521 10.1016/s0028-3932(98)00145-6

[39] Boyd LA, Wintein CJ. FAPTA2. explicit information interferes with implicit motor learning of both continuous and discrete movement tasks after stroke. J Neurol Phys Ther. 2006;30(2):46–57. 16796767 10.1097/01.npt.0000282566.48050.9b

[40] Rosso C, Lamy JC. Does resting motor threshold predict motor hand recovery after stroke? Front Neurol. 2018;9:1020. 30555404 10.3389/fneur.2018.01020 PMC6281982

[41] Kemlin C, Moulton E, Lamy J-C, et al Elucidating the structural and functional correlates of upper-limb poststroke motor impairment. Stroke. 2019;50(12):3647–3649. 31645211 10.1161/STROKEAHA.119.027126

[42] Daghsen L, Checkouri T, Wittwer A, et al The relationship between corticospinal excitability and structural integrity in stroke patients. J Neurol Neurosurg Psychiatry. 2024;96(1):85–94. 39242199 10.1136/jnnp-2023-331996

[43] Spampinato DA, Ibanez J, Rocchi L, Rothwell J. Motor potentials evoked by transcranial magnetic stimulation: Interpreting a simple measure of a complex system. J Physiol. 2023;601(14):2827–2851. 37254441 10.1113/JP281885 PMC10952180

[44] Butefisch CM, Netz J, Wessling M, Seitz RJ, Homberg V. Remote changes in cortical excitability after stroke. Brain. 2003;126(Pt 2):470–481. 12538413 10.1093/brain/awg044

[45] Butefisch CM, Wessling M, Netz J, Seitz RJ, Homberg V. Relationship between interhemispheric inhibition and motor cortex excitability in subacute stroke patients. Neurorehabil Neural Repair. 2008;22(1):4–21. 17507644 10.1177/1545968307301769

[46] Bestmann S, Krakauer JW. The uses and interpretations of the motor-evoked potential for understanding behaviour. Exp Brain Res. 2015;233(3):679–689. 25563496 10.1007/s00221-014-4183-7

[47] Li Voti P, Conte A, Suppa A, et al Correlation between cortical plasticity, motor learning and BDNF genotype in healthy subjects. Exp Brain Res. 2011;212(1):91–99. 21537966 10.1007/s00221-011-2700-5

[48] Rosenkranz K, Kacar A, Rothwell JC. Differential modulation of motor cortical plasticity and excitability in early and late phases of human motor learning. J Neurosci. 2007;27(44):12058–12066. 17978047 10.1523/JNEUROSCI.2663-07.2007 PMC6673358

[49] Perez MA, Lungholt BKS, Nyborg K, Nielsen JB. Motor skill training induces changes in the excitability of the leg cortical area in healthy humans. Exp Brain Res. 2004;159(2):197–205. 15549279 10.1007/s00221-004-1947-5

[50] Wadden KP, Woodward TS, Metzak PD, et al Compensatory motor network connectivity is associated with motor sequence learning after subcortical stroke. Behav Brain Res. 2015;286:136–145. 25757996 10.1016/j.bbr.2015.02.054 PMC4390540

[51] Makin TR, Krakauer JW. Against cortical reorganisation. Elife. 2023;12:e84716. 37986628 10.7554/eLife.84716 PMC10662956

[52] Ziemann U, Tergau F, Wischer S, Hildebrandt J, Paulus W. Pharmacological control of facilitatory I-wave interaction in the human motor cortex. A paired transcranial magnetic stimulation study. Electroencephalogr Clin Neurophysiol. 1998;109:321–330. 9751295 10.1016/s0924-980x(98)00023-x

[53] Ziemann U . Modulation of practice-dependent plasticity in human motor cortex. Brain. 2001;124(6):1171–1181. 11353733 10.1093/brain/124.6.1171

[54] Grigoras IF, Stagg CJ. Recent advances in the role of excitation-inhibition balance in motor recovery post-stroke. Fac Rev. 2021;10:58. 34308424 10.12703/r/10-58 PMC8265564

[55] Clarkson AN, Huang BS, Macisaac SE, Mody I, Carmichael ST. Reducing excessive GABA-mediated tonic inhibition promotes functional recovery after stroke. Nature. 2010;468(7321):305–309. 21048709 10.1038/nature09511 PMC3058798

[56] Garry MI, Thomson RHS. The effect of test TMS intensity on short-interval intracortical inhibition in different excitability states. Exp Brain Res. 2009;193(2):267–274. 18974984 10.1007/s00221-008-1620-5

[57] Paparella I, Vanderwalle G, Stagg CJ, Maquet P. An integrated measure of GABA to characterize post-stroke plasticity. Neuroimage Clin. 2023;39:103463. 37406594 10.1016/j.nicl.2023.103463 PMC10339061

[58] Muellbacher W, Richards C, Ziemann U, et al Improving hand function in chronic stroke. Arch Neurol. 2002;59(8):1278. 12164724 10.1001/archneur.59.8.1278

[59] Richards LG, Stewart KC, Woodbury ML, Senesac C, Cauraugh JH. Movement-dependent stroke recovery: A systematic review and meta-analysis of TMS and fMRI evidence. Neuropsychologia. 2008;46(1):3–11. 17904594 10.1016/j.neuropsychologia.2007.08.013 PMC2248459

[60] Hildesheim FE, Silver AN, Dominguez-Vargas AU, et al Predicting individual treatment response to rTMS for motor recovery after stroke: A review and the CanStim perspective. Front Rehabil Sci. 2022;3:795335. 36188894 10.3389/fresc.2022.795335 PMC9397689

[61] Edwards JD, Dominguez-Vargas AU, Rosso C, et al A translational roadmap for transcranial magnetic and direct current stimulation in stroke rehabilitation: Consensus-based core recommendations from the third stroke recovery and rehabilitation roundtable. Int J Stroke. 2024;19(2):145–157. 37824726 10.1177/17474930231203982 PMC10811969

[62] Feng W, Wang J, Chhatbar PY, et al Corticospinal tract lesion load: An imaging biomarker for stroke motor outcomes. Ann Neurol. 2015;78(6):860–870. 26289123 10.1002/ana.24510 PMC4715758

[63] Ferris JK, Lo BP, Barisano G, et al Modulation of the association between corticospinal tract damage and outcome after stroke by White matter hyperintensities. Neurol. 2024;102(10):e209387. 10.1212/WNL.0000000000209387 PMC11196095 38701386

[64] Julkunen P, Saisanen L, Hukkanen T, Danner N, Kononen M. Does second-scale intertrial interval affect motor evoked potentials induced by single-pulse transcranial magnetic stimulation? Brain Stimul. 2012;5(4):526–532. 21962979 10.1016/j.brs.2011.07.006

[65] Pellicciari MC, Miniussi C, Ferrari C, Koch G, Bortoletto M. Ongoing cumulative effects of single TMS pulses on corticospinal excitability: An intra- and inter-block investigation. Clin Neurophysiol. 2016;127(1):621–628. 25823698 10.1016/j.clinph.2015.03.002

[66] Kujirai T, Caramia MD, Rothwell JC, et al Corticocortical inhibition in human motor cortex. J Physiol. 1993;471:501–519. 8120818 10.1113/jphysiol.1993.sp019912 PMC1143973

[67] Peurala SH, Muller-Dahlhaus JF, Arai N, Ziemann U. Interference of short-interval intracortical inhibition (SICI) and short-interval intracortical facilitation (SICF). Clin Neurophysiol. 2008;119(10):2291–2297. 18723394 10.1016/j.clinph.2008.05.031

[68] Ni Z, Chen R. Short-interval intracortical inhibition: A complex measure. Clin Neurophysiol. 2008;119(10):2175–2176. 18752992 10.1016/j.clinph.2008.06.007

[69] Stinear CM, Barber PA, Smale PR, Coxon JP, Fleming MK, Byblow WD. Functional potential in chronic stroke patients depends on corticospinal tract integrity. Brain. 2006;130(1):170–180. 10.1093/brain/awl333 17148468

[70] Robertson EM, Pascual-Leone A, Miall RC. Current concepts in procedural consolidation. Nat Rev Neurosci. 2004;5(7):576–582. 15208699 10.1038/nrn1426

[71] Kantak SS, Winstein CJ. Learning-performance distinction and memory processes for motor skills: A focused review and perspective. Behav Brain Res. 2012;228(1):219–231. 22142953 10.1016/j.bbr.2011.11.028

[72] De Las Heras B, Cristini J, Rodrigues L, et al Lesion location influences motor skill retention in subacute stroke. In: Abstracts of the European Stroke Organization Conference, Basel; 2024.

[73] Stinear CM . Prediction of motor recovery after stroke: Advances in biomarkers. Lancet Neurol. 2017;16(10):826–836. 28920888 10.1016/S1474-4422(17)30283-1

