# Supplementary material for: Lesion location changes the association between brain excitability and the performance of a short-term visuomotor adaptation task post-stroke
Source: Brain Commun. 2025 Oct 31;7(6):fcaf430. doi: 10.1093/braincomms/fcaf430 (PMC12628505; doi:10.1093/braincomms/fcaf430)
Supplement: fcaf430_Supplementary_Data [file fcaf430_supplementary_data.pdf]

Supplementary Material

Supplementary Table 1. Demographic and clinical data for the cortical group and subcortical group with cortico-spinal tract (CST) unaffected and subcortical group with CST affected.

	Cortical	Subcortical		p value
		CST unaffected	CST affected	
**N**	34	43	26	
**Age (y)**	64.79 (11.61)	64.90 (10.16)	65.88 (8.46)	0.906
**Sex (F/M)**	7/27	17/26	8/18	0.196
**Subacute/Chronic**	28/6	25/18	16/10	0.063
**Days since stroke**	135.94 (199.85)	331.69 (456.45)	287.73 (389.01)	0.068
**Subacute**	63.64 (22.85)	62.72 (20.03)	69.31 (27.45)	0.644
**Chronic**	473.33 (308.08)	705.27 (509.91)	673.20 (445.64)	0.575
**Type of stroke (I/H)**	33/1	35/8	25/1	0.0330*
**NIHSS**	1.79 (1.73)	1.88 (2.10)	2.11 (2.10)	0.819
**MoCA**	23.75 (4.95)	22.42 (5.21)	25.38 (3.83)	0.052
**$CMSA_{arm}$**	6.54 (1.09)	5.82 (1.63)	4.76 (1.87)$^{\#\#}$	0.0002*
**$CMSA_{hand}$**	6.42 (0.87)	5.67 (1.67)	4.76 (2.08)$^{\#\#\#\#}$	0.0007*
**$MVC_{affected}$**	0.79 (0.25)	0.67 (0.32)	0.54 (0.25)$^{\#}$	0.0060*
**$MVC_{unaffected}$**	0.86 (0.21)	0.82 (0.31)	0.84 (0.24)	0.843
**$BBT_{affected}$**	49.57 (11.44)	41.17 (15.11)	40.56 (15.98)$^{\#\#\#}$	0.0203*
**$BBT_{unaffected}$**	52.12 (7.74)	48.38 (9.54)	51.88 (9.65)	0.144

BBT, Box and Block Test; CMSA, Chedoke-McMaster Stroke Assessment; CST, corticospinal tract; H, hemorrhagic; I, ischemic; M, Male; MoCA, Montreal Cognitive Assessment; MVC, Maximal voluntary contraction; NIHSS, National Institutes of Health Stroke Scale; F, Female. Values are presented as means and standard deviations (SD). *Analysis of variance showed significant differences between groups ($p<0.05$). $^{\#}$CST affected is significantly lower than the cortical group. $^{\#\#}$CST affected is significantly lower than the two other lesion groups. $^{\#\#\#}$ Both CST unaffected and CST affected are significantly lower than the cortical group. $^{\#\#\#\#}$All lesion groups differ statistically from each other.

Supplementary Table 2.1. Adjusted linear mixed models for motor skill performance comparing cortical and subcortical groups.

Motor skill performance	DFNum	DFDen	F ratio	p value
**Lesion Location (2 groups)**	1	88.7	0.81	0.370
**Block**	3	169.9	29.02	0.0001*
**Lesion Location*Block**	3	169.9	1.26	0.287
**Age**	1	86.2	9.15	0.0033*
**Days since stroke**	1	86.2	3.53	0.063
**NIHSS**	1	86.2	9.69	0.0025*
**$MVC_{affected}$**	1	86.3	0.03	0.847

MVC, Maximal voluntary contraction; NIHSS, National Institutes of Health Stroke Scale. * $p<0.05$.

Supplementary Table 2.2. Unadjusted linear mixed models for motor skill performance comparing cortical and subcortical groups.

Motor skill perfromance	DFNum	DFDen	F ratio	p value
**Lesion Location (2 groups)**	2	99.6	0.62	0.430
**Block**	3	188.7	30.25	0.0001*
**Lesion Location*Block**	3	188.7	1.09	0.353

* $p<0.05$.

Supplementary Table 2.3. Adjusted linear mixed models for motor skill performance comparing cortical group, subcortical group with cortico-spinal tract (CST) unaffected and subcortical group with CST affected.

Motor skill performance	DFNum	DFDen	F ratio	p value
**Lesion Location (3 groups)**	2	87.8	0.57	0.564
**Block**	3	167.2	26.85	0.0001*
**Lesion Location*Block**	6	193.3	0.69	0.652
**Age**	1	85.2	9.16	0.0033*
**Days since stroke**	1	85.1	3.66	0.059
**NIHSS**	1	85.1	9.20	0.0032*
**$MVC_{affected}$**	1	85.3	0.005	0.938

MVC, Maximal voluntary contraction; NIHSS, National Institutes of Health Stroke Scale. * $p<0.05$.

Supplementary Table 2.4. Unadjusted linear mixed models for motor skill performance comparing cortical group and subcortical group with cortico-spinal tract (CST) unaffected and subcortical group with CST affected.

Motor skill performance	DFNum	DFDen	F ratio	p value
**Lesion Location (3 groups)**	2	98.6	0.38	0.684
**Block**	3	185.2	28.49	0.0001*
**Lesion Location*Block**	6	212.7	0.82	0.552

* $p<0.05$.

Supplementary Table 3. Adjusted linear mixed models for different cortico-spinal excitability (CSE) measures in ipsilesional and contralesional hemispheres comparing cortical and subcortical groups.

	DFNum	DFDen	F ratio	p value
**RMT ipsilesional**				
Lesion Location (2 groups)	1	84	3.75	0.056
Age	1	84	0.14	0.703
Days since stroke	1	84	0.20	0.648
NIHSS	1	84	0.01	0.919
**Resting MEP ipsilesional**				
Lesion Location (2 groups)	1	83	2.39	0.125
Age	1	83	0.05	0.816
Days since stroke	1	83	0.01	0.891
NIHSS	1	83	0.25	0.612
**Active MEP ipsilesional**				
Lesion Location (2 groups)	1	83	4.78	0.031*
Age	1	83	0.06	0.794
Days since stroke	1	83	4.97	0.028*
NIHSS	1	83	5.42	0.022*
**CSP ipsilesional**				
Lesion Location (2 groups)	1	81	0.0005	0.982
Age	1	81	0.05	0.812
Days since stroke	1	81	0.20	0.651
NIHSS	1	81	8.82	0.003*
**ICF ipsilesional**				
Lesion Location (2 groups)	1	80	0.82	0.366
Age	1	80	0.0005	0.980
Days since stroke	1	80	0.32	0.572
NIHSS	1	80	2.24	0.137
**SICI ipsilesional**				
Lesion Location (2 groups)	1	73	1.52	0.220
Age	1	73	0.001	0.965
Days since stroke	1	73	0.02	0.874
NIHSS	1	73	1.59	0.210
**RMT contralesional**				
Lesion Location (2 groups)	1	93	0.02	0.878
Age	1	93	0.02	0.868
Days since stroke	1	93	0.02	0.874
NIHSS	1	93	1.33	0.251
**Resting MEP contralesional**				
Lesion Location (2 groups)	1	90	1.07	0.303
Age	1	90	1.22	0.272
Days since stroke	1	90	0.72	0.397
NIHSS	1	90	0.01	0.889
**Active MEP contralesional**				
Lesion Location (2 groups)	1	92	0.08	0.772
Age	1	92	0.87	0.352
Days since stroke	1	92	1.18	0.278
NIHSS	1	92	0.63	0.428
**CSP contralesional**				
Lesion Location (2 groups)	1	91	1.31	0.255
Age	1	91	4.15	0.044*
Days since stroke	1	91	2.51	0.116
NIHSS	1	91	0.13	0.711

**ICF contralesional**				
Lesion Location (2 groups)	1	89	0.32	0.569
Age	1	89	0.006	0.934
Days since stroke	1	89	0.26	0.608
NIHSS	1	89	0.12	0.728
**SICI contralesional**				
Lesion Location (2 groups)	1	81	2.20	0.141
Age	1	81	0.35	0.552
Days since stroke	1	81	0.06	0.804
NIHSS	1	81	1.02	0.314

CSP, cortical silent period; CST, corticospinal tract; ICF, intracortical facilitation; MEP, motor evoked potential; RMT, resting motor threshold; SICI, short-intracortical inhibition. * p<0.05.

Supplementary Table 4. Cortico-spinal excitability (CSE) measures in ipsilesional and contralesional hemispheres for cortical group and subcortical group with cortico-spinal tract (CST) unaffected and CST affected.

	**Cortical**	**Subcortical**		**p value**
		**CST unaffected**	**CST affected**	
**Ipsilesional**				
**RMT (%)**	48.84 (2.19)	53.26 (1.96)	55.87 (2.70)	0.119
**Resting MEP (mV)**	0.51 (0.06)	0.46 (0.05)	0.19 (0.08)[##]	0.0091*
**Active MEP (mV)**	1.74 (0.14)	1.57 (0.12)	0.85 (0.17)[##]	0.0005*
**CSP (ms)**	0.19 (0.009)	0.18 (0.008)	0.19 (0.01)	0.753
**ICF (%)**	171.51 (20.27)	172 (19.84)	226.79 (32.60)	0.180
**SICI (%)**	93.18 (11.27)	77.32 (9.09)	68.34 (11.71)	0.432
**Contralesional**				
**RMT (%)**	51.74 (1.92)	51.72 (1.68)	50.81 (2.15)	0.935
**Resting MEP (mV)**	0.47 (0.04)	0.43 (0.03)	0.37 (0.04)	0.382
**Active MEP (mV)**	1.70 (0.14)	1.86 (0.12)	1.59 (0.16)	0.387
**CSP (ms)**	0.17 (0.004)	0.17 (0.004)	0.16 (0.005)	0.282
**ICF (%)**	173.35 (23.46)	188 (23.05)	189.85 (29.33)	0.851
**SICI (%)**	54.93 (16.38)	86.67 (15.66)	76.56 (22.67)	0.319

CSP, cortical silent period; CST, corticospinal tract; ICF, intracortical facilitation; MEP, motor evoked potential; RMT, resting motor threshold; SICI, short-intracortical inhibition. Values are means estimates and standard errors of the mean (SEM). LMMs were adjusted for stroke severity, age and time since stroke * p<0.05.
[##]CST affected is significantly lower than the two other lesion groups.

Supplementary Table 5. Adjusted linear mixed models for different cortico-spinal excitability (CSE) measures in ipsilesional and contralesional hemispheres comparing the cortical group, and subcortical groups with cortico-spinal tract (CST) unaffected and CST affected.

	DFNum	DFDen	F ratio	p value
**RMT ipsilesional**				
Lesion Location (3 groups)	2	83	2.17	0.119
Age	1	83	0.15	0.692
Days since stroke	1	83	0.19	0.656
NIHSS	1	83	0.01	0.907
**Resting MEP ipsilesional**				
Lesion Location (3 groups)	2	82	4.91	0.0091*
Age	1	82	0.08	0.772
Days since stroke	1	82	0.01	0.888
NIHSS	1	82	0.19	0.657
**Active MEP ipsilesional**				
Lesion Location (3 groups)	2	82	8.29	0.0005*
Age	1	82	0.04	0.828
Days since stroke	1	82	5.59	0.020*
NIHSS	1	82	5.60	0.020*
**CSP ipsilesional**				
Lesion Location (3 groups)	2	80	0.28	0.753
Age	1	80	0.07	0.791
Days since stroke	1	80	0.20	0.652
NIHSS	1	80	8.70	0.004*
**ICF ipsilesional**				
Lesion Location (3 groups)	2	79	1.75	0.180
Age	1	79	0.00003	0.995
Days since stroke	1	79	0.32	0.573
NIHSS	1	79	2.05	0.156
**SICI ipsilesional**				
Lesion Location (3 groups)	2	72	0.84	0.432
Age	1	72	0.005	0.943
Days since stroke	1	72	0.03	0.856
NIHSS	1	72	1.53	0.219
**RMT contralesional**				
Lesion Location (3 groups)	2	92	0.06	0.935
Age	1	92	0.03	0.856
Days since stroke	1	92	0.02	0.869
NIHSS	1	92	1.28	0.260
**Resting MEP contralesional**				
Lesion Location (3 groups)	2	89	0.97	0.382
Age	1	89	1.32	0.253
Days since stroke	1	89	0.67	0.414
NIHSS	1	89	0.04	0.834
**Active MEP contralesional**				
Lesion Location (3 groups)	2	91	0.95	0.387
Age	1	91	0.73	0.392
Days since stroke	1	91	1.28	0.260
NIHSS	1	91	0.54	0.464
**CSP contralesional**				
Lesion Location (3 groups)	2	90	1.28	0.282
Age	1	90	4.44	0.037*
Days since stroke	1	90	2.32	0.130

NIHSS	1	90	0.15	0.696
**ICF contralesional**				
Lesion Location (3 groups)	2	88	0.16	0.851
Age	1	88	0.006	0.934
Days since stroke	1	88	0.26	0.610
NIHSS	1	88	0.11	0.730
**SICI contralesional**				
Lesion Location (3 groups)	2	80	1.16	0.316
Age	1	80	0.31	0.575
Days since stroke	1	80	0.06	0.806
NIHSS	1	80	1.003	0.319

CSP, cortical silent period; CST, corticospinal tract; ICF, intracortical facilitation; MEP, motor evoked potential; RMT, resting motor threshold; SICI, short-intracortical inhibition. * $p<0.05$.

Supplementary Table 6. Primary multivariate linear regression analysis investigating the associations between motor skill performance and cortico-spinal excitability (CSE) in the ipsilesional hemisphere for cortical and subcortical groups.

	**Cortical**			**Subcortical**		
**Ipsilesional**	**Estimate (95% CI)**	**p value**	R^2	**Estimate (95% CI)**	**p value**	R^2
**RMT (%)**	-0.25 (-0.47, -0.03)	0.024*	0.42	-0.03 (-0.08, 0.16)	0.527	0.23
Age	-0.26 (-0.45, -0.07)	0.008*		-0.12 (-0.29, 0.05)	0.162	
Days since stroke	-0.0005 (-0.01, 0.009)	0.906		0.001 (-0.002, 0.005)	0.500	
NIHSS	-0.02 (-1.21, 1.17)	0.971		-0.76 (-1.66, 0.12)	0.090	
$MVC_{affected}$	2.16 (-7.32, 11.65)	0.643		3.86 (-2.48, 0.20)	0.227	
**Resting MEP (mV)**	2.91 (-3.09, 8.92)	0.327	0.31	-4.46 (-8.54, -0.36)	0.033*	0.30
Age	-0.27 (-0.47, -0.06)	0.013*		-0.12 (-0.28, 0.04)	0.135	
Days since stroke	-0.003 (-0.01, 0.007)	0.517		0.001 (-0.002, 0.005)	0.437	
NIHSS	0.12 (-1.17, 1.41)	0.846		-0.75 (-1.63, 0.11)	0.088	
$MVC_{affected}$	5.50 (-4.25, 15.25)	0.256		4.42 (-1.91, 10.76)	0.167	
**Active MEP (mV)**	1.44 (-1.35, 4.24)	0.298	0.32	-1.66 (-3.71, 0.38)	0.109	0.27
Age	-0.26 (-0.47, -0.06)	0.013*		-0.13 (-0.30, 0.03)	0.112	
Days since stroke	-0.002 (-0.01, 0.008)	0.679		0.0003 (-0.003, 0.004)	0.853	
NIHSS	0.29 (-1.04, 1.64)	0.652		-0.88 (-1.76, 0.005)	0.051	
$MVC_{affected}$	4.33 (-6.18, 14.85)	0.404		4.14 (-2.36, 10.66)	0.206	
**CSP (ms)**	-68.23 (-150.80, 14.33)	0.101	0.36	-1.15 (-28.40, 26.09)	0.932	0.26
Age	-0.24 (-0.44, -0.04)	0.019*		-0.14 (-0.31, 0.03)	0.113	
Days since stroke	-0.00 (-0.01, 0.007)	0.597		-3e-4 (-0.004, 0.003)	0.869	
NIHSS	0.53 (-0.82, 1.88)	0.425		-1.11 (-2.09, -0.13)	0.026*	
$MVC_{affected}$	0.12 (-12.03, 12.27)	0.984		2.69 (-3.74, 9.12)	0.404	
**ICF**	-1.93 (-4.83, 0.97)	0.184	0.34	1.34 (0.14, 2.54)	0.028*	0.32
Age	-0.24 (-0.44, -0.04)	0.021*		-0.08 (-0.25, 0.08)	0.324	
Days since stroke	-0.002 (-0.01, 0.009)	0.747		0.001 (-0.002, 0.004)	0.465	
NIHSS	0.38 (-0.95, 1.73)	0.556		-0.93 (-1.79, -0.06)	0.036*	
$MVC_{affected}$	7.05 (-2.15, 16.25)	0.127		4.59 (-1.66, 10.86)	0.146	
**SICI**	-3.93 (-6.89, -0.98)	0.011*	0.44	3.45 (0.34, 6.56)	0.030*	0.31
Age	-0.25 (-0.45, -0.06)	0.011*		-0.11 (-0.29, 0.06)	0.214	
Days since stroke	-0.002 (-0.01, 0.008)	0.740		0.001 (-0.002, 0.005)	0.447	
NIHSS	0.40 (-0.82, 1.63)	0.502		-0.96 (-1.86, -0.07)	0.035*	
$MVC_{affected}$	2.83 (-6.88, 12.55)	0.552		4.73 (-1.70, 11.18)	0.145	

CSP, cortical silent period; ICF, intracortical facilitation; MEP, motor evoked potential; RMT, resting motor threshold; SICI, short-intracortical inhibition. * $p<0.05$.

Supplementary Table 7. Primary multivariate linear regression analysis investigating the associations between motor skill performance and cortico-spinal excitability (CSE) in the contralesional hemisphere for cortical and subcortical groups.

	Cortical			Subcortical		
**Contralesional**	**Estimate (95% CI)**	**p value**	**R^2**	**Estimate (95% CI)**	**p value**	**R^2**
**RMT (%)**	-0.14 (-0.32, 0.02)	0.087	0.36	-0.006 (-0.15, 0.14)	0.933	0.25
Age	-0.23 (-0.42, -0.05)	0.013*		-0.14 (-0.28, 0.006)	0.059	
Days since stroke	-0.002 (-0.01, 0.008)	0.607		0.001 (-0.002, 0.005)	0.518	
NIHSS	-0.008 (-1.22, 1.20)	0.988		-1.001 (-1.83, -0.17)	0.019*	
$MVC_{affected}$	5.63 (-2.84, 14.11)	0.183		2.68 (-2.91, 8.28)	0.340	
**Resting MEP (mV)**	-1.26 (-10.25, 7.72)	0.774	0.28	-2.10 (-8.61, 4.40)	0.519	0.26
Age	-0.22 (-0.43, -0.01)	0.033*		-0.13 (-0.28, 0.02)	0.088	
Days since stroke	-0.003 (-0.01, 0.007)	0.502		0.001 (-0.002, 0.005)	0.421	
NIHSS	-0.06 (-1.59, 1.46)	0.928		-1.01 (-1.91, -0.10)	0.029*	
$MVC_{affected}$	8.29 (-0.88, 17.47)	0.074		3.10 (-2.77, 8.97)	0.294	
**Active MEP (mV)**	0.25 (-2.98, 3.48)	0.874	0.29	0.79 (-1.03, 2.61)	0.389	0.26
Age	-0.23 (-0.43, -0.04)	0.017*		-0.14 (-0.28, 0.008)	0.064	
Days since stroke	-0.003 (-0.01, 0.007)	0.566		0.001 (-0.002, 0.005)	0.434	
NIHSS	0.19 (-1.16, 1.54)	0.770		-1.04 (-1.88, -0.20)	0.015*	
$MVC_{affected}$	7.51 (-1.17, 16.20)	0.087		2.23 (-3.46, 7.94)	0.435	
**CSP (ms)**	-49.84 (-146.52, 46.83)	0.299	0.30	3.71 (-55.06, 62.50)	0.899	0.31
Age	-0.22 (-0.41, -0.02)	0.025*		-0.14 (-0.29, 0.01)	0.068	
Days since stroke	-0.001 (-0.01, 0.009)	0.743		0.0006 (-0.003, 0.004)	0.724	
NIHSS	0.42 (-0.92, 1.76)	0.525		-1.08 (-1.92, -0.24)	0.012*	
$MVC_{affected}$	5.80 (-3.34, 14.94)	0.203		2.75 (-2.90, 8.41)	0.333	
**ICF**	0.58 (-1.91, 3.07)	0.636	0.29	-0.10 (-1.17, 0.95)	0.838	0.26
Age	-0.23 (-0.43, -0.04)	0.017*		-0.12 (-0.28, 0.02)	0.103	
Days since stroke	-0.003 (-0.01, 0.007)	0.482		0.001 (-0.002, 0.004)	0.519	
NIHSS	0.05 (-1.27, 1.39)	0.928		-1.06 (-1.91, -0.22)	0.014*	
$MVC_{affected}$	7.36 (-1.30, 16.03)	0.092		3.08 (-2.80, 8.97)	0.297	
**SICI**	-1.83 (-8.11, 4.43)	0.550	0.24	0.44 (-1.21, 2.10)	0.592	0.26
Age	-0.22 (-0.43, -0.02)	0.031*		-0.13 (-0.31, 0.05)	0.153	
Days since stroke	-0.002 (-0.01, 0.009)	0.664		0.001 (-0.002, 0.005)	0.418	
NIHSS	0.15 (-1.19, 1.49)	0.814		-0.91 (-1.84, 0.01)	0.054	
$MVC_{affected}$	7.12 (-3.10, 17.36)	0.163		3.85 (-2.44, 10.15)	0.224	

CSP, cortical silent period; ICF, intracortical facilitation; MEP, motor evoked potential; RMT, resting motor threshold; SICI, short-intracortical inhibition. * $p<0.05$.

Supplementary Table 8. Multivariate linear regression analysis testing the interaction of lesion location (cortical vs subcortical) on the association between cortico-spinal excitability (CSE) measures and motor skill performance in the ipsilesional and contralesional hemispheres.

	**Ipsilesional**			**Contralesional**		
**Ipsilesional**	**Estimate (95% CI)**	**p value**	**R^2**	**Estimate (95% CI)**	**p value**	**R^2**
**RMT (%)**	-0.11 (-0.22, 0.004)	0.060	0.30	-0.09 (-0.19, 0.02)	0.113	0.29
Age	-0.19 (-0.31, -0.06)	0.004*		-0.18 (-0.28, -0.06)	0.002*	
Days since stroke	0.001 (-0.002, 0.004)	0.594		0.001 (-0.003, 0.004)	0.660	
NIHSS	-0.52 (-1.20, 0.16)	0.134		-0.64 (-1.30, 0.01)	0.054	
$MVC_{affected}$	3.66 (-1.34, 8.67)	0.149		4.08 (-0.37, 8.54)	0.072	
Location (2 groups)	0.41 (-0.89, 1.73)	0.530		0.79 (-0.47, 2.06)	0.216	
Location*RMT	-0.14 (-0.25, -0.03)	0.013*		-0.09 (-0.19, 0.02)	0.113	
**Resting MEP (mV)**	-1.02 (-4.44, 2.38)	0.551	0.29	-1.92 (-6.96, 3.12)	0.451	0.26
Age	-0.19 (-0.31, -0.06)	0.004*		-0.16 (-0.28, -0.04)	0.007*	
Days since stroke	0.0008 (-0.003, 0.004)	0.651		0.001 (-0.003, 0.004)	0.591	
NIHSS	-0.40 (-1.09, 0.29)	0.252		-0.61 (-1.33, 0.11)	0.095	
$MVC_{affected}$	5.32 (0.22, 10.42)	0.041*		5.33 (0.70, 9.95)	0.024*	
Location (2 groups)	0.60 (-0.71, 1.92)	0.363		0.63 (-0.69, 1.97)	0.344	
Location*Resting MEP	3.48 (0.18, 6.79)	0.039*		0.53 (-4.57, 5.64)	0.835	
**Active MEP (mV)**	-0.36 (-1.95, 1.22)	0.647	0.26	0.20 (-1.48, 1.89)	0.810	0.25
Age	-0.19 (-0.32, -0.06)	0.003*		-0.17 (-0.29, -0.06)	0.003*	
Days since stroke	-0.0001 (-0.003, 0.003)	0.963		0.0009 (-0.003, 0.004)	0.626	
NIHSS	-0.46 (-1.16, 0.23)	0.192		-0.56 (-1.24, 0.11)	0.102	
$MVC_{affected}$	5.17 (-0.09, 10.43)	0.054		4.71 (0.14, 9.29)	0.043*	
Location (2 groups)	0.54 (-0.79, 1.89)	0.420		0.72 (-0.58, 2.03)	0.275	
Location*Active MEP	1.35 (-0.11, 2.81)	0.069		-0.32 (-2.02, 1.38)	0.707	
**CSP (ms)**	-20.41 (-53.06, 12.23	0.217	0.27	-13.83 (-62.98, 35.33)	0.577	0.27
Age	-0.191 (-0.32, -0.06)	0.005*		-0.17 (-0.29, -0.05)	0.005*	
Days since stroke	-0.001 (-0.004, 0.003)	0.763		0.0003 (-0.003, 0.003)	0.865	
NIHSS	-0.55 (-1.30, 0.20)	0.148		-0.55 (-1.23, 0.12)	0.107	
$MVC_{affected}$	3.82 (-1.48, 9.13)	0.156		4.74 (0.18, 9.29)	0.041*	
Location (2 groups)	0.79 (-0.56, 2.15)	0.248		0.77 (-0.53, 2.08)	0.240	
Location*CSP	-14.96 (-46.90, 16.98)	0.354		-23.19 (-72.20, 25.83)	0.350	
**ICF**	-0.18 (-1.59, 1.23)	0.797	0.30	0.36 (-0.84, 1.57)	0.546	0.26
Age	-0.16 (-0.28, -0.03)	0.015*		-0.17 (-0.28, -0.05)	0.005*	
Days since stroke	0.0009 (-0.003, 0.004)	0.619		0.0006 (-0.003, 0.004)	0.730	
NIHSS	-0.42 (-1.13, 0.28)	0.235		-0.63 (-1.32, 0.05)	0.070	
$MVC_{affected}$	6.03 (0.97, 11.09)	0.020*		5.10 (0.38, 9.81)	0.034*	
Location (2 groups)	0.66 (-0.66, 1.99)	0.324		0.85 (-0.47, 2.17)	0.204	
Location*ICF	-1.48 (-2.90, -0.06)	0.041*		0.39 (-0.84, 1.63)	0.529	
**SICI**	-0.10 (-2.20, 2.002)	0.924	0.32	-0.74 (-3.59, 2.11)	0.606	0.25
Age	-0.18 (-0.31, -0.05)	0.007*		-0.17 (-0.30, -0.04)	0.009*	
Days since stroke	0.0009 (-0.003, 0.004)	0.608		0.001 (-0.002, 0.004)	0.540	
NIHSS	-0.48 (-1.18, 0.21)	0.174		-0.46 (-1.18, 0.25)	0.203	
$MVC_{affected}$	4.99 (-0.21, 10.19)	0.059		5.60 (0.58, 10.63)	0.029*	
Location (2 groups)	0.89 (-0.48, 2.27)	0.201		0.58 (-0.91, 2.07)	0.441	
Location*SICI	-3.35 (-5.42, -1.29)	0.001*		-1.39 (-4.29, 1.51)	0.342	

CSP, cortical silent period; ICF, intracortical facilitation; MEP, motor evoked potential; RMT, resting motor threshold; SICI, short-intracortical inhibition. * $p<0.05$.

Supplementary Table 9. Exploratory multivariate linear regression analysis investigating the associations between motor skill performance and cortico-spinal excitability (CSE) in the ipsilesional hemisphere for cortical, subcortical CST unaffected and CST affected groups.

	Cortical			Subcortical					
				CST unaffected			CST affected		
Ipsilesional	Estimate (95% CI)	p value	R^2	Estimate (95% CI)	p value	R^2	Estimate (95% CI)	p value	R^2
**RMT (%)**	-0.25 (-0.47, -0.03)	0.024*	0.42	0.09 (-0.05, 0.24)	0.217	0.30	-0.06 (-0.38, 0.25)	0.662	0.21
Age	-0.26 (-0.45, -0.07)	0.008*		-0.12 (-0.32, 0.07)	0.205		-0.14 (-0.61, 0.31)	0.500	
Days since stroke	-0.0005 (-0.01, 0.009)	0.906		0.0006 (-0.003, 0.004)	0.746		0.004 (-0.008, 0.01)	0.485	
NIHSS	-0.02 (-1.21, 1.17)	0.971		-0.68 (-1.70, 0.32)	0.176		-1.41 (-4.06, 1.23)	0.267	
$MVC_{affected}$	2.16 (-7.32, 11.65)	0.643		4.27 (-2.68, 11.23)	0.219		0.69 (-19.28, 20.68)	0.940	
**Resting MEP (mV)**	2.91 (-3.09, 8.92)	0.327	0.31	-4.009 (-8.43, 0.41)	0.074	0.33	-17.57 (-41.55, 6.40)	0.135	0.36
Age	-0.27 (-0.47, -0.06)	0.013*		-0.11 (-0.31, 0.07)	0.231		-0.26 (-0.69, 0.17)	0.212	
Days since stroke	-0.003 (-0.01, 0.007)	0.517		0.0008 (-0.003, 0.004)	0.666		0.004 (-0.006, 0.01)	0.347	
NIHSS	0.12 (-1.17, 1.41)	0.846		-0.58 (-1.58, 0.41)	0.239		-1.87 (-4.29, 0.54)	0.116	
$MVC_{affected}$	5.50 (-4.25, 15.25)	0.256		5.59 (-1.39, 12.59)	0.112		-2.57 (-20.83, 15.68)	0.762	
**Active MEP (mV)**	1.44 (-1.35, 4.24)	0.298	0.32	-2.37 (-4.77, 0.01)	0.051	0.35	0.93 (-8.27, 10.14)	0.827	0.22
Age	-0.26 (-0.47, -0.06)	0.013*		-0.14 (-0.33, 0.05)	0.145		-0.11 (-0.57, 0.34)	0.592	
Days since stroke	-0.002 (-0.01, 0.008)	0.679		-0.0003 (-0.004, 0.003)	0.874		0.004 (-0.008, 0.01)	0.438	
NIHSS	0.29 (-1.04, 1.64)	0.652		-0.61 (-1.60, 0.36)	0.207		-1.40 (-4.58, 1.77)	0.352	
$MVC_{affected}$	4.33 (-6.18, 14.85)	0.404		6.09 (-0.93, 13.12)	0.086		-1.12 (-21.39, 19.15)	0.905	
**CSP (ms)**	-68.23 (-150.80, 14.33)	0.101	0.36	-5.41 (-35.67, 24.85)	0.717	0.29	37.29 (-68.22, 142.80)	0.449	0.31
Age	-0.24 (-0.44, -0.04)	0.019*		-0.16 (-0.37, 0.05)	0.139		-0.17 (-0.63, 0.27)	0.397	
Days since stroke	-0.00 (-0.01, 0.007)	0.597		-0.0004 (-0.005, 0.004)	0.837		0.004 (-0.01, 0.01)	0.546	
NIHSS	0.53 (-0.82, 1.88)	0.425		-0.83 (-1.95, 0.29)	0.141		-2.42 (-5.51, 0.65)	0.110	
$MVC_{affected}$	0.12 (-12.03, 12.27)	0.984		3.64 (-3.48, 10.77)	0.305		-1.98 (-22.37, 18.39)	0.832	
**ICF**	-1.93 (-4.83, 0.97)	0.184	0.34	0.47 (-1.14, 2.09)	0.555	0.31	2.64 (0.34, 4.94)	0.027*	0.50
Age	-0.24 (-0.44, -0.04)	0.021*		-0.07 (-0.29, 0.13)	0.467		-0.10 (-0.45, 0.25)	0.541	
Days since stroke	-0.002 (-0.01, 0.009)	0.747		0.0005 (-0.003, 0.004)	0.774		0.006 (-0.003, 0.01)	0.166	
NIHSS	0.38 (-0.95, 1.73)	0.556		-0.80 (-1.83, 0.23)	0.123		-1.35 (-3.47, 0.76)	0.187	
$MVC_{affected}$	7.05 (-2.15, 16.25)	0.127		5.44 (-1.69, 12.58)	0.129		5.98 (-11.29, 23.26)	0.461	
**SICI**	-3.93 (-6.89, -0.98)	0.011*	0.44	2.33 (-1.14, 5.82)	0.179	0.34	10.31 (2.07, 18.54)	0.019*	0.56
Age	-0.25 (-0.45, -0.06)	0.011*		-0.07 (-0.28, 0.12)	0.439		-0.19 (-0.60, 0.21)	0.304	
Days since stroke	-0.002 (-0.01, 0.008)	0.740		0.0005 (-0.003, 0.004)	0.774		0.01 (-0.001, 0.02)	0.071	
NIHSS	0.40 (-0.82, 1.63)	0.502		-0.82 (-1.83, 0.17)	0.103		-1.83 (-4.20, 0.52)	0.111	
$MVC_{affected}$	2.83 (-6.88, 12.55)	0.552		5.86 (-1.11, 12.83)	0.096		7.53 (-10.70, 25.77)	0.374	

CSP, cortical silent period; CST, corticospinal tract; ICF, intracortical facilitation; MEP, motor evoked potential; RMT, resting motor threshold; SICI, short-intracortical inhibition. * $p<0.05$.

Supplementary Table 10. Exploratory multivariate linear regression analysis investigating the associations between motor skill performance and cortico-spinal excitability (CSE) in the contralesional hemisphere for cortical, subcortical CST unaffected and CST affected groups.

	Cortical			Subcortical					
				CST unaffected			CST affected		
Contralesional	Estimate (95% CI)	p value	R^2	Estimate (95% CI)	p value	R^2	Estimate (95% CI)	p value	R^2
**RMT (%)**	-0.14 (-0.32, 0.02)	0.087	0.36	0.05 (-0.12, 0.23)	0.560	0.27	-0.13 (-0.42, 0.15)	0.347	0.34
Age	-0.23 (-0.42, -0.05)	0.013*		-0.12 (-0.29, 0.04)	0.135		-0.21 (-0.56, 0.14)	0.225	
Days since stroke	-0.002 (-0.01, 0.008)	0.607		0.0004 (-0.003, 0.004)	0.805		0.005 (-0.004, 0.01)	0.252	
NIHSS	-0.008 (-1.22, 1.20)	0.988		-0.75 (-1.71, 0.20)	0.119		-2.09 (-4.12, -0.06)	0.044*	
$MVC_{affected}$	5.63 (-2.84, 14.11)	0.183		3.50 (-2.99, 10.00)	0.280		-2.09 (-16.10, 11.91)	0.756	
**Resting MEP (mV)**	-1.26 (-10.25, 7.72)	0.774	0.28	-0.72 (-8.66, 7.20)	0.852	0.26	-4.79 (-19.41, 9.83)	0.498	0.33
Age	-0.22 (-0.43, -0.01)	0.033*		-0.11 (-0.29, 0.05)	0.179		-0.16 (-0.53, 0.19)	0.341	
Days since stroke	-0.003 (-0.01, 0.007)	0.502		0.0005 (-0.003, 0.004)	0.791		0.005 (-0.004, 0.01)	0.227	
NIHSS	-0.06 (-1.59, 1.46)	0.928		-0.83 (-1.92, 0.25)	0.127		-1.70 (-3.73, 0.32)	0.094	
$MVC_{affected}$	8.29 (-0.88, 17.47)	0.074		3.77 (-3.44, 11.00)	0.294		-0.30 (-13.79, 13.18)	0.962	
**Active MEP (mV)**	0.25 (-2.98, 3.48)	0.874	0.29	0.85 (-1.28, 3.005)	0.420	0.28	-0.64 (-5.57, 4.29)	0.787	0.31
Age	-0.23 (-0.43, -0.04)	0.017*		-0.11 (-0.28, 0.05)	0.164		-0.18 (-0.55, 0.17)	0.287	
Days since stroke	-0.003 (-0.01, 0.007)	0.566		0.0007 (-0.003, 0.004)	0.703		0.005 (-0.004, 0.01)	0.242	
NIHSS	0.19 (-1.16, 1.54)	0.770		-0.87 (-1.88, 0.13)	0.086		-1.94 (-4.07, 0.19)	0.071	
$MVC_{affected}$	7.51 (-1.17, 16.20)	0.087		2.90 (-3.88, 9.68)	0.390		-0.36 (-14.17, 13.43)	0.955	
**CSP (ms)**	-49.84 (-146.52, 46.83)	0.299	0.30	-13.37 (-84.73, 57.98)	0.705	0.27	67.19 (-70.44, 204.82)	0.316	0.39
Age	-0.22 (-0.41, -0.02)	0.025*		-0.11 (-0.29, 0.05)	0.185		-0.23 (-0.59, 0.11)	0.175	
Days since stroke	-0.001 (-0.01, 0.009)	0.743		0.0006 (-0.003, 0.004)	0.745		0.004 (-0.006, 0.01)	0.387	
NIHSS	0.42 (-0.92, 1.76)	0.525		-0.78 (-1.76, 0.19)	0.114		-2.10 (-4.12, -0.08)	0.042*	
$MVC_{affected}$	5.80 (-3.34, 14.94)	0.203		3.65 (-2.97, 10.29)	0.269		-1.53 (-15.04, 11.97)	0.812	
**ICF**	0.58 (-1.91, 3.07)	0.636	0.29	0.11 (-1.23, 1.46)	0.863	0.30	-0.94 (-3.11, 1.22)	0.368	0.34
Age	-0.23 (-0.43, -0.04)	0.017*		-0.10 (-0.28, 0.07)	0.237		-0.21 (-0.56, 0.14)	0.224	
Days since stroke	-0.003 (-0.01, 0.007)	0.482		0.0005 (-0.003, 0.004)	0.775		0.005 (-0.004, 0.01)	0.240	
NIHSS	0.05 (-1.27, 1.39)	0.928		-0.83 (-1.81, 0.14)	0.092		-2.23 (-4.38, -0.08)	0.042*	
$MVC_{affected}$	7.36 (-1.30, 16.03)	0.092		4.83 (-1.95, 11.62)	0.156		-3.36 (-18.67, 11.93)	0.648	
**SICI**	-1.83 (-8.11, 4.43)	0.550	0.24	0.83 (-1.35, 3.02)	0.441	0.31	-0.16 (-3.28, 2.96)	0.912	0.33
Age	-0.22 (-0.43, -0.02)	0.031*		-0.08 (-0.29, 0.13)	0.429		-0.26 (-0.69, 0.16)	0.202	
Days since stroke	-0.002 (-0.01, 0.009)	0.664		0.0009 (-0.003, 0.005)	0.629		0.006 (-0.004, 0.01)	0.233	
NIHSS	0.15 (-1.19, 1.49)	0.814		-0.61 (-1.70, 0.47)	0.258		-2.05 (-4.37, 0.25)	0.077	
$MVC_{affected}$	7.12 (-3.10, 17.36)	0.163		6.005 (-1.49, 13.50)	0.112		-0.23 (-15.26, 14.78)	0.973	

CSP, cortical silent period; CST, corticospinal tract; ICF, intracortical facilitation; MEP, motor evoked potential; RMT, resting motor threshold; SICI, short-intracortical inhibition. * $p<0.05$.

Supplementary Table 11. Sensitivity multivariate linear regression analysis investigating the associations between motor skill performance and cortico-spinal excitability (CSE) in the ipsilesional hemisphere for pure cortical (excluding cortico-subcortical lesions) and subcortical groups.

	**Cortical**			**Subcortical**		
**Ipsilesional**	**Estimate (95% CI)**	**p value**	R^2	**Estimate (95% CI)**	**p value**	R^2
**RMT (%)**	-0.27 (-0.63, 0.08)	0.117	0.51	-0.03 (-0.08, 0.16)	0.527	0.23
Age	-0.38 (-0.72, -0.05)	0.028*		-0.12 (-0.29, 0.05)	0.162	
Days since stroke	-0.002 (-0.01, 0.01)	0.771		0.001 (-0.002, 0.005)	0.500	
NIHSS	0.304 (-2.09, 2.70)	0.787		-0.76 (-1.66, 0.12)	0.090	
$MVC_{affected}$	3.69 (-17.10, 24.49)	0.705		3.86 (-2.48, 0.20)	0.227	
**Resting MEP (mV)**	4.28 (-5.87, 14.43)	0.376	0.43	-4.46 (-8.54, -0.36)	0.033*	0.30
Age	-0.36 (-0.73, 0.002)	0.051		-0.12 (-0.28, 0.04)	0.135	
Days since stroke	-0.005 (-0.02, 0.009)	0.432		0.001 (-0.002, 0.005)	0.437	
NIHSS	-0.10 (-2.59, 2.37)	0.925		-0.75 (-1.63, 0.11)	0.088	
$MVC_{affected}$	7.13 (-15.37, 29.64)	0.502		4.42 (-1.91, 10.76)	0.167	
**Active MEP (mV)**	1.27 (-3.00, 5.55)	0.529	0.41	-1.66 (-3.71, 0.38)	0.109	0.27
Age	-0.34 (-0.71, 0.02)	0.063		-0.13 (-0.30, 0.03)	0.112	
Days since stroke	-0.005 (-0.02, 0.01)	0.516		0.0003 (-0.003, 0.004)	0.853	
NIHSS	0.01 (-2.64, 2.68)	0.987		-0.88 (-1.76, 0.005)	0.051	
$MVC_{affected}$	8.25 (-15.22, 31.73)	0.458		4.14 (-2.36, 10.66)	0.206	
**CSP (ms)**	-48.97 (-186.70, 88.76)	0.453	0.42	-1.15 (-28.40, 26.09)	0.932	0.26
Age	-0.32 (-0.67, 0.03)	0.071		-0.14 (-0.31, 0.03)	0.113	
Days since stroke	-0.005 (-0.02, 0.01)	0.490		-3e-4 (-0.004, 0.003)	0.869	
NIHSS	0.27 (-2.61, 3.16)	0.839		-1.11 (-2.09, -0.13)	0.026*	
$MVC_{affected}$	4.52 (-24.40, 33.45)	0.739		2.69 (-3.74, 9.12)	0.404	
**ICF**	-3.31 (-7.38, 0.75)	0.101	0.52	1.34 (0.14, 2.54)	0.028*	0.32
Age	-0.32 (-0.64, -0.002)	0.048*		-0.08 (-0.25, 0.08)	0.324	
Days since stroke	-0.003 (-0.01, 0.01)	0.635		0.001 (-0.002, 0.004)	0.465	
NIHSS	0.29 (-2.06, 2.65)	0.790		-0.93 (-1.79, -0.06)	0.036*	
$MVC_{affected}$	10.70 (-6.86, 28.27)	0.209		4.59 (-1.66, 10.86)	0.146	
**SICI**	-5.68 (-10.02, -1.34)	0.015*	0.64	3.45 (0.34, 6.56)	0.030*	0.31
Age	-0.30 (-0.60, -0.004)	0.047*		-0.11 (-0.29, 0.06)	0.214	
Days since stroke	-0.004 (-0.01, 0.009)	0.509		0.001 (-0.002, 0.005)	0.447	
NIHSS	-0.37 (-2.54, 1.78)	0.707		-0.96 (-1.86, -0.07)	0.035*	
$MVC_{affected}$	5.93 (-15.05, 26.93)	0.542		4.73 (-1.70, 11.18)	0.145	

CSP, cortical silent period; ICF, intracortical facilitation; MEP, motor evoked potential; RMT, resting motor threshold; SICI, short-intracortical inhibition. * $p<0.05$.

Supplementary Table 12. Sensitivity multivariate linear regression analysis investigating the associations between motor skill performance and cortico-spinal excitability (CSE) in the contralesional hemisphere for pure cortical (excluding cortico-subcortical lesions) and subcortical groups.

	Cortical			Subcortical		
**Contralesional**	**Estimate (95% CI)**	**p value**	**R^2**	**Estimate (95% CI)**	**p value**	**R^2**
**RMT (%)**	-0.20 (-0.46, 0.06)	0.120	0.50	-0.006 (-0.15, 0.14)	0.933	0.25
Age	-0.32 (-0.60, -0.03)	0.029*		-0.14 (-0.28, 0.006)	0.059	
Days since stroke	-0.004 (-0.01, 0.008)	0.458		0.001 (-0.002, 0.005)	0.518	
NIHSS	-0.29 (-2.51, 1.91)	0.777		-1.001 (-1.83, -0.17)	0.019*	
$MVC_{affected}$	11.67 (-3.62, 26.97)	0.123		2.68 (-2.91, 8.28)	0.340	
**Resting MEP (mV)**	4.67 (-12.55, 21.90)	0.568	0.40	-2.10 (-8.61, 4.40)	0.519	0.26
Age	-0.32 (-0.65, 0.006)	0.054		-0.13 (-0.28, 0.02)	0.088	
Days since stroke	-0.005 (-0.02, 0.009)	0.426		0.001 (-0.002, 0.005)	0.421	
NIHSS	0.10 (-2.61, 2.81)	0.937		-1.01 (-1.91, -0.10)	0.029*	
$MVC_{affected}$	14.02 (-2.35, 30.39)	0.087		3.10 (-2.77, 8.97)	0.294	
**Active MEP (mV)**	0.70 (-4.47, 5.88)	0.773	0.39	0.79 (-1.03, 2.61)	0.389	0.26
Age	-0.28 (-0.59, 0.01)	0.064		-0.14 (-0.28, 0.008)	0.064	
Days since stroke	-0.006 (-0.02, 0.009)	0.397		0.001 (-0.002, 0.005)	0.434	
NIHSS	-0.09 (-2.75, 2.57)	0.943		-1.04 (-1.88, -0.20)	0.015*	
$MVC_{affected}$	13.62 (-2.94, 30.19)	0.099		2.23 (-3.46, 7.94)	0.435	
**CSP (ms)**	-57.56 (-197.26, 82.13)	0.390	0.42	3.71 (-55.06, 62.50)	0.899	0.31
Age	-0.25 (-0.56, 0.05)	0.097		-0.14 (-0.29, 0.01)	0.068	
Days since stroke	-0.004 (-0.01, 0.01)	0.548		0.0006 (-0.003, 0.004)	0.724	
NIHSS	-0.006 (-2.43, 2.42)	0.995		-1.08 (-1.92, -0.24)	0.012*	
$MVC_{affected}$	11.22 (-6.03, 28.49)	0.184		2.75 (-2.90, 8.41)	0.333	
**ICF**	0.04 (-3.71, 3.81)	0.978	0.39	-0.10 (-1.17, 0.95)	0.838	0.26
Age	-0.28 (-0.59, 0.02)	0.068		-0.12 (-0.28, 0.02)	0.103	
Days since stroke	-0.006 (-0.02, 0.009)	0.391		0.001 (-0.002, 0.004)	0.519	
NIHSS	-0.25 (-2.80, 2.29)	0.835		-1.06 (-1.91, -0.22)	0.014*	
$MVC_{affected}$	13.76 (-3.24, 30.76)	0.104		3.08 (-2.80, 8.97)	0.297	
**SICI**	-2.67 (-12.38, 7.04)	0.557	0.39	0.44 (-1.21, 2.10)	0.592	0.26
Age	-0.30 (-0.65, 0.04)	0.081		-0.13 (-0.31, 0.05)	0.153	
Days since stroke	-0.005 (-0.02, 0.01)	0.464		0.001 (-0.002, 0.005)	0.418	
NIHSS	-0.16 (-2.92, 2.58)	0.896		-0.91 (-1.84, 0.01)	0.054	
$MVC_{affected}$	15.26 (-7.96, 38.48)	0.176		3.85 (-2.44, 10.15)	0.224	

CSP, cortical silent period; ICF, intracortical facilitation; MEP, motor evoked potential; RMT, resting motor threshold; SICI, short-intracortical inhibition. * $p<0.05$.
